# Transposable elements contribute to fungal genes and impact fungal lifestyle

**DOI:** 10.1038/s41598-019-40965-0

**Published:** 2019-03-13

**Authors:** Anna Muszewska, Kamil Steczkiewicz, Marta Stepniewska-Dziubinska, Krzysztof Ginalski

**Affiliations:** 1 Institute of Biochemistry and Biophysics, Polish Academy of Sciences, Pawinskiego 5A, 02-106 Warsaw, Poland; 2 Laboratory of Bioinformatics and Systems Biology, CeNT, University of Warsaw, Zwirki i Wigury 93, 02-089 Warsaw, Poland

## Abstract

The last decade brought a still growing experimental evidence of mobilome impact on host’s gene expression. We systematically analysed genomic location of transposable elements (TEs) in 625 publicly available fungal genomes from the NCBI database in order to explore their potential roles in genome evolution and correlation with species’ lifestyle. We found that non-autonomous TEs and remnant copies are evenly distributed across genomes. In consequence, they also massively overlap with regions annotated as genes, which suggests a great contribution of TE-derived sequences to host’s coding genome. Younger and potentially active TEs cluster with one another away from genic regions. This non-randomness is a sign of either selection against insertion of TEs in gene proximity or target site preference among some types of TEs. Proteins encoded by genes with old transposable elements insertions have significantly less repeat and protein-protein interaction motifs but are richer in enzymatic domains. However, genes only proximal to TEs do not display any functional enrichment. Our findings show that adaptive cases of TE insertion remain a marginal phenomenon, and the overwhelming majority of TEs are evolving neutrally. Eventually, animal-related and pathogenic fungi have more TEs inserted into genes than fungi with other lifestyles. This is the first systematic, kingdom-wide study concerning mobile elements and their genomic neighbourhood. The obtained results should inspire further research concerning the roles TEs played in evolution and how they shape the life we know today.

## Introduction

Transposable elements (TEs) constitute a significant but understudied fraction of eukaryotic genomes. They are mobile genetic units that proliferate and expand to distant genomic regions. TEs are classified into two classes based on their transposition mechanism. Class I groups elements that transpose using an RNA intermediate, whereas Class II members skip RNA transcript and transpose directly from DNA to DNA^1^. TE landscape of most eukaryotic genomes consists of Class I representatives including: retrotransposons with Long Terminal Repeats (LTR retrotransposons), Long Interspersed Nuclear Elements (LINEs), Short Interspersed Nuclear Elements (SINEs), as well as of Class II DNA transposons that encode a classic DDE transposase (“cut and paste” DNA TEs, TIRs) or comply with yet unknown mechanism of transposition, e.g. Helitrons and Polintons/Mavericks.

For a long time, transposable elements were considered just another species of “junk DNA” and the hypothesis on their regulatory roles raised by Barbara McClintock^2^ remained ignored. Their impact on eukaryotic evolution and genome function is still a matter of vigorous debate between two extremes: TEs as passive genetic material for selection on one side and powerful factors that immediately impact cell and organism’s fate on the other^3,4^. Nonetheless, TEs can be considered as molecular parasites, which introduce mutations and eventually contribute significantly to genome size inflation^5–7^. Like other parasites, they take part in an arms race against host’s defence mechanisms and organisms have developed multiple complex mechanisms to keep their genomes clear from foreign DNA. The most common are DNA methylation^8^, targeting by tRNA-derived small RNAs^9–11^, RNAi mediated silencing^12^ and repeat-induced point mutations^13^.

TE insertion breaks continuity of co-selected traits, alters gene transcription, leads to chromosomal rearrangements by promoting recombination^14,15^ and promotes insertional mutations, which can impose deleterious consequences for target loci^4^. In the last decade, remarkable examples of TE functional impact on host, mostly animal, have been described, including organ development^16^, karyotype changes^17^, cell fate regulation^18^ and stress response modulation^19^. TE-derived genes play crucial roles in all living organisms and massively alter expression of the proximal genes^20^. A TE can modify host transcripts *via* exonisation of itself, induction of original exon skipping what leads to alternative transcripts, insertion into an ORF (into an existing frame) creating a new fusion protein, and insertion of alternative polyadenylation signals. It can also interfere with gene regulation by delivering novel, illegitimate promoter sequences. For example, a single transposon-derived protein, CSB-PGBD3 (domesticated transposase) can interact with as many as 900 remnant TE sequences and plays roles in gene regulation upon DNA damage^21^. Also, host’s protein-coding mRNAs can be occasionally retrotransposed by retrotransposon-related machinery, which can result in formation of novel pseudogenes and genes^22^. The latter might eventually donate polyadenylation sites to neighbouring genes and further expand transcript diversity^22^.

Phenomena resulting from gene-transposable element proximity have been thoroughly studied mainly for model animals^20,23,24^ and plants^3^, and only a few studies included fungal genomes despite genomic resource abundance^25–27^. For instance, a remnant LTR retrotransposon insertion into promoter region of a gene coding for MFS1 transporter was found to induce this gene overexpression and to enhance fungicide resistance^28^. Also, gene clusters can be regulated by neighbouring TEs, e.g. the penicillin cluster in *Aspergillus nidulans* has lower expression in the absence of Pbla element^29^. In *Schizosaccharomyces pombe*, Tf1 element has a preference for promoters of stress-related genes, which eventually enhances their expression and promotes survival of the fungus^30^.

TE neighbourhood within a window of 1 kb has a repressive effect on neighbouring genes in fungi equipped with functional methylation machinery, but casts no such effect in *Saccharomyces cerevisiae*, which lacks methylation^25^. Genes within 1 kb to a Gypsy or hAT transposon have lower expression in *Coccidioides immitis*^31^. In this organism, TEs are often inserted in proximity of phosphorylation-related genes. Castanera and colleagues showed also that the presence of TE clusters has more pronounced regulatory effects on gene expression as compared to a single TE upstream or downstream^25^.

Some fungal pathogens of plants have genomes with a clearly dualistic architecture described by the two-speed model of evolution. The core genome is densely packed with housekeeping genes while a lifestyle-adapting part contains effector genes and TEs^7,32^. This genome architecture was reported for versatile fungal pathogens among them *Fusarium*^33^, *Leptosphaeria*^34^ and *Verticillium*^35^. The lifestyle-specific genome is expected to be enriched in TEs, as they may play roles in host switching and adaptation to new ecological niches^36^, which can be observed in *Magnaporthe oryzae*, where genes associated with TEs are involved in host specialization^37^. In consequence, even closely related fungal taxa may differ significantly in transposable content, e.g. *Amanita* species with saprophytic and mycorrhizal lifestyles^38^.

Encouraged by the aforementioned experimental screenings demonstrating the impact of TEs on gene expression, we performed a systematic analysis of their genomic context in publicly available fungal genomes. Here, we investigate the immediate neighbourhood of transposable elements, with special focus on co-localizing genes. Moreover, we interpret our results from a lifestyle perspective.

## Methods

### Genomes and transposable elements

Fungal proteomes were downloaded from NCBI on 17th August 2016^39^ and genomic sequences were downloaded from NCBI genome portal on 18th of August 2016. 625 genomic assemblies with corresponding proteomes analysed in this study are listed in Supplementary Table S1. Genome sequences deposited at the NCBI were obtained using diverse sequencing techniques, with different sequencing depths, assembled and annotated using a plethora of approaches. In consequence, there ought to be gene calling inconsistencies, missing genome fragments and to deal with it our study will focus on general trends instead of singularities. Genomic coordinates of TEs were inferred in the course of *de novo* and homology-based TE annotation. Irf inverted repeat finder^40^ (irf parameters used: matching weight 2, mismatching weight 3, indel penalty 5, match probability 80, indel probability 10, minimum alignment score to report 20, maximum stem length to report 500000, MaxLoop 10000, additional options: -a3 -t4 1000 -t5 5000) and RepeatModeler^41^ were used to detect TE candidates *de novo*. Irf hits were classified using the RepeatModeler annotating script. Multiple overlapping hits were removed by clustering with RepBase database entries^42^ using CD-HIT^43^, and the resulting sequence dataset of TE consensus sequences was used as a library for RepeatMasker homology search^44^ (RepeatMasker was invoked with options: -gccalc -no_is, TEs with scores above 200 were taken). All the resulting sequences were scanned with manually curated list of reference Pfam HMM profiles (using pfam_scan.pl with E-value threshold 0.01)^45^ and CDD profiles (RPS-BLAST with E-value threshold 0.001)^46^ listed in Supplementary Table S2. This TE annotation pipeline has been successfully employed previously in the study of DNA TE’s^47^ as well as in a growing number of genome annotation studies^48–50^. The chosen protein domains are either associated with TE activity or related to TEs and were collected based on TE architectures known from RepBase and literature. The elements containing sequences similar to known TE-related domains are labelled along the manuscript as “with domain” transposable elements. Sequences without detectable similarity to known TE domains were considered as fragments and remnants of old TEs. A schematic workflow of the analyses is shown as Supplementary Fig. S1.

### Neighbourhood classification

Three classes of TE neighbours were defined: (i) nothing, (ii) other TE and (iii) gene. In order to provide a robust and consistent neighbourhood classification, we defined the following set of rules. First of all, to adhere to varying genome architectures for each species, an adaptive scanning window size was estimated as a median of gene distances in the whole assembly (Supplementary Table S1) with the top size of 1 kb. The minimal median gene distance value was 71 for *Enterocytozoon bieneusi* and maximum 8,997 for *Edhazardia aedis*. In total, 12 analysed assemblies had the window narrower than 100 bp while 79 - wider than 1 kb. All protein sequences encoded by genes partially overlapping with TE coordinates were scanned against a list of TE-related protein domains using pfam_scan.pl tool. If the gene had no detectable TE-related domains, the TE borders were shortened and the gene became TE’s immediate neighbour; otherwise, the gene was included into TE’s borders and the neighbourhood was determined against expanded TE coordinates. If TE fully covered any gene, it replaced this gene in further neighbourhood assessment. Moreover, if the neighbouring gene contained an inner TE, which was also located within the window distance to the analysed TE, this inner element was annotated as a neighbour (Supplementary Fig. S2). When two or more TEs overlapped, they were merged together and tagged with the most specific annotation common to all of participating TEs. When merged TEs were of totally distinct species, the newly defined TE was tagged as a ‘composite’.

A TE inserted into a gene can reside within a 3′ UTR, 5′ UTR, intron or exon. Unfortunately, the majority of analysed assemblies lacked gene inner structures and even less included UTRs at all. In consequence, we were not able to study the detailed location of TEs at a sub-genic level.

The encoded proteins were scanned for secretion signals using TargetP^51^ and were assigned to GO categories using pfam2GO table^52^.

### Data analysis

All genomes with incomplete annotation, for instance without gene predictions, were excluded from analysis, as mentioned above. Genome statistics (size, density, intron per gene) were computed based on the assembly sequences and gff annotation files downloaded from the NCBI database. Since gene calling strategies vary in reliability between genomes and initial data quality directly impacts our neighbourhood analyses, we have selected only highly significant patterns emerging from analyses described in this manuscript.

Information on fungal lifestyles, as in our previous study on DNA transposons^47^, was derived from the available literature. Categories including host type (plant, animal, fungus), main habitat (soil/dung, water) and lifestyle (pathogenic, symbiotic and saprotrophic) were assigned to every species in the dataset. Noteworthy, a single fungus could represent multiple categories, if applicable, e.g. species functioning both as a plant symbiont and animal pathogen (see Supplementary Table 1). Taxonomical annotation was derived from the NCBI taxonomy database, with manual curation when needed (see Supplementary Table 1). TE types were described using a 2-level hierarchy comprising Wicker’s orders/Repbase classes (e.g. LINEs, SINEs, LTRs) and superfamilies (e.g. Copia, hAT).

Exploratory analysis and basic statistics for the dataset were carried out using pandas and seaborn Python packages. Statistical tests were performed in Python with the scipy package. Distributions of distances between TEs and genes for fungi with different lifestyles were compared with Mann-Whitney U test. Relationships between the number of TE inserted into genes as well as other genome statistics were evaluated using McFadden’s R-squared for logistic regression with binomial errors. The logistic regression models were built with statsmodels package. Enrichment analyses were performed using binomial distribution, and upper-bounds for p-values were computed with formula derived from Hoeffding’s inequality:

$$p \mbox{-} value\le exp(\frac{-2{(np-k)}^{2}}{n}),$$

where n is the number of trials, k is the number of successes and p is the success probability.

The genome features are available as Supplementary Table 1 and the code for statistical procedures is available as a python code in a Jupyter Notebook (Supplementary File 1)^53^.

## Results

### TE fragments contribute to fungal genes

2,023,812 TE fragments and 293,746 TEs with a TE-related protein domain were identified in 625 fungal genomes (Supplementary Table S2 lists protein domains either associated with TE activity or related to TEs). Our TE counts are likely to be underestimated and there are two major reasons for that. The first and more fundamental one is a derivative of methods used in whole genome sequencing projects, which rely mostly on sequencing reads of lengths insufficient for effective reconstitution of long repeat regions. The second reason lies in our approach, as we chose to apply rather stringent filtering of identified TE fragments in order to increase the method’s reliability. All TE candidates had to be confirmed with RepeatMasker using extended fragments library as described in Methods section. Additionally, all TEs regarded as still functional were supposed to contain at least one known TE-related protein domain.

Fungal genomes have different gene densities and architectures ranging from very compact in endoparasitic Microsporidia to relatively big and complex *Tuber* and *Puccinia* genomes. A question arises whether and how such rough genome characteristics correlate with TE localization in different taxa.

**Figure 1:**
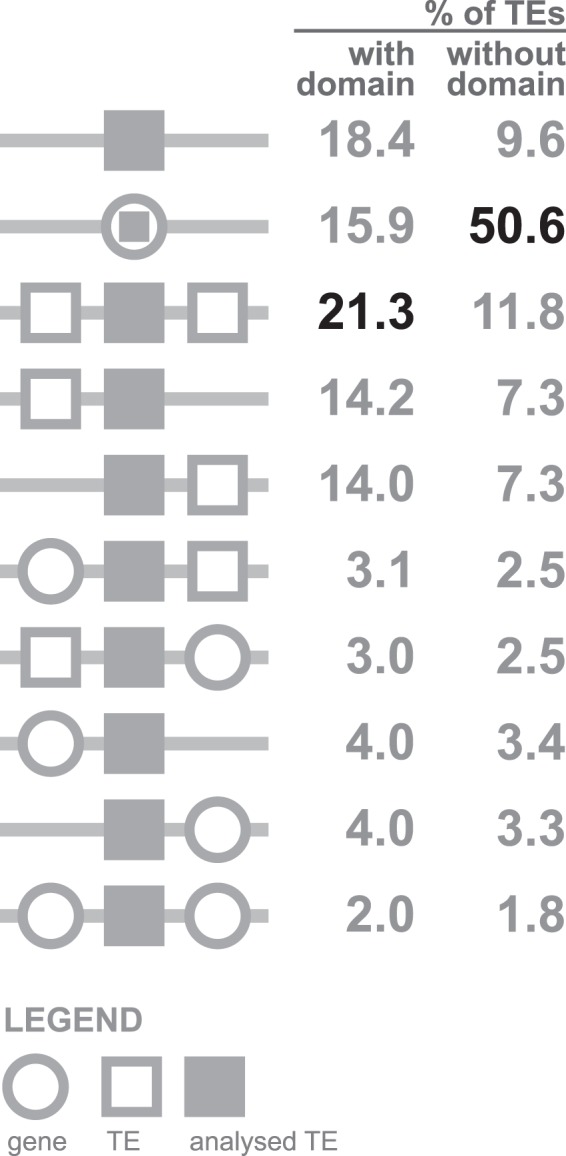
Schematic representation of TE location in analysed fungal assemblies.

**Figure 2:**
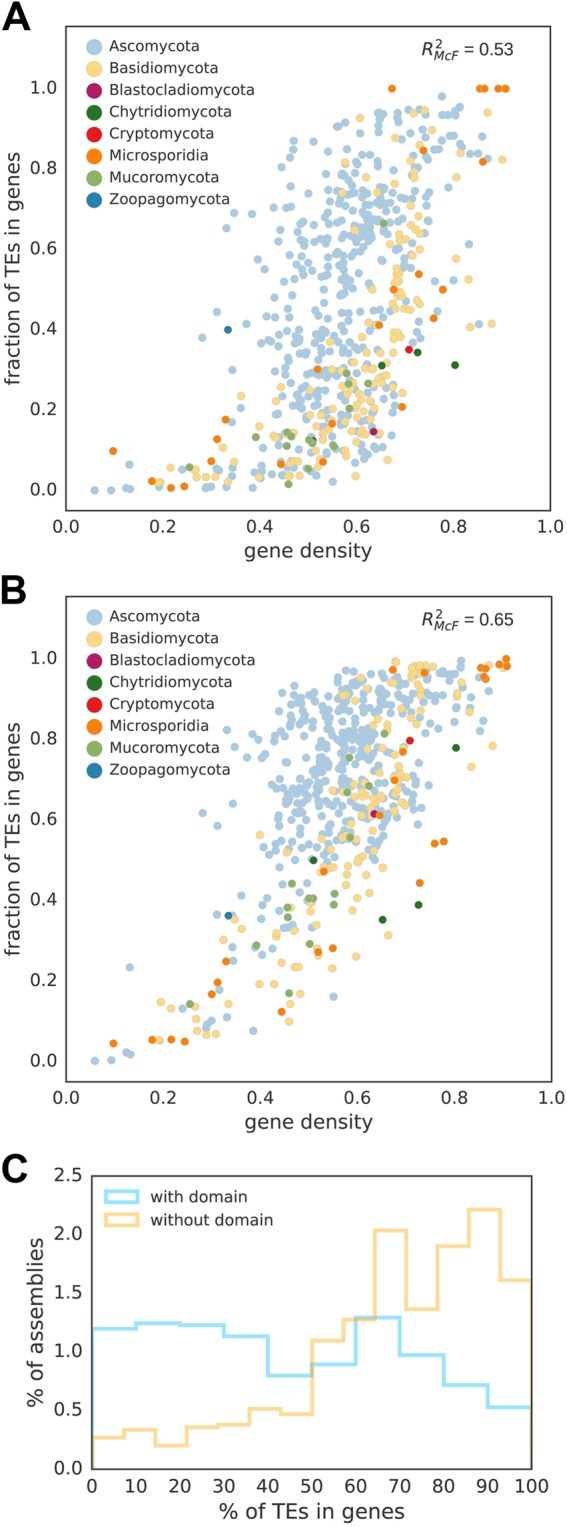
Distribution of in-gene TEs with (**A**) and without (**B**) TE-related domains in relation to genomes with different gene density. (**C**) Distribution of genome assemblies with different fractions of TEs located in genes.

We found that non-autonomous TEs and remnants massively overlap with regions annotated as genes. These results suggest a great contribution of TE-derived sequences to host’s genes (Fig. 1). 50.6% of non-autonomous TEs are inserted into a genic region (1,024,918) and 11.6% (235,593) TEs fragments were found in proximity of gene on either side, being equally ubiquitous downstream and upstream of genes (116,722 downstream, 118,871 upstream). The location of a TE fragment between two genes is relatively rare (1.8% of TEs, 36,841). That totals to 64% of non-autonomous TEs co-localising with genes and points at the compact architecture of many fungal genomes assuming random distribution of ancient TEs and genes in many of the fungal genomes. More compact genomes host more remnant TEs inserted into genes as compared to genomes with greater non-genic space (Fig. 2A). 14.6% of TEs had another TE as a neighbour either upstream (147,874) or downstream (147,114), 11.8% (238,024) TEs were located in-between other TEs, while 9.6% of TEs fragments (193,448) had neither genes nor TEs identified within the chosen scanning window. In total, 35.9% of analysed TEs either had another TEs as exclusive neighbours or lacked neighbourhood at all.

### Active TEs cluster with other TEs

Transposable elements with at least one protein domain typical for mobile elements have a distinct genomic neighbourhood profile. They are rarely found within or in close proximity of genes (less than 15.9%, 46,789 of these elements are inserted into a gene and 16,2%, 47,493 are close to a gene) and tend to cluster with other TEs (almost 49.5%, 145,384) or locate in regions without genes and other TEs (18.4%, 54,080). Academ is an exception here because most of their copies contain recognizable protein domains themselves (81%, 1,568/1,938) and are classified as overlapping with a gene. These domains are encoded by a TE but are not classified as TE-specific (e.g. DEAD/DEAH box helicase domain (PFAM: PF00270) Replication, recombination and repair, recQ_fam (CDD:129701)), which hacks classification criteria and eventually makes Academs frequently annotated as host genes.

This non-random distribution of potentially active TEs might be a sign of general negative selection imposed on TEs interfering with gene coding regions or target site preference as observed for some types of TEs (e.g. Zisupton^54^). Even if insertion preference might play a pivotal role in shaping the genomic landscape of active elements, once they became inactivated, the evolutionary pressure against them faded and TE fragments have survived in genomic areas where active TEs are not allowed.

### Genome properties and TE localization

There is a significant correlation (R^2^_McF_ = 0.53 for TEs with a domain and R^2^_McF_ = 0.65 for TE fragments) between the fraction of TEs targeting genes and genome compactness measured as fraction of the genome occupied by genes (Fig. 2A,B). The smaller the gene distances and fewer non-genic regions, the more TE-related sequences overlap with genes likely as a result of scarcity of other genomic locations.

Overall ubiquity of remnant TEs in gene neighbourhood can be a consequence of the random distribution of TEs resulting from neutrality of old and fragmented TEs, lack of traceable target site preference among most types of TEs, and most probably recurrent usage of ancient TE-derived sequences. Interestingly, we observe a binomial distribution of in-gene insertion frequency for TEs with TE-related domains (Fig. 2C). The two peaks correspond to two distinct genome architectures within fungi: one with a higher fraction of both remnant and coding TEs in genes (mostly in Saccharomycotina, see Supplementary Fig. S3) and the other one with only remnant TE debris located within genes (filamentous fungi). The former TE distribution is peculiar and might be a consequence of Saccharomycotina’s selection on compactness of the genomes.

### Remnant TEs populate enzyme-encoding genes

#### Non-autonomous TEs and TE remnants

Protein-coding genes impacted by old TE insertions are significantly depleted in protein repeat motifs such as Ankyrin, WD40 and in protein-protein interaction domains like F-box (See Supplementary Table 3). This pattern has not been described so far and will be explored in detail in further studies. One might expect that repeat sequences will appear as artefacts with *de novo* TE searches mainly due to large families present in a single genome. However, the obtained result showing protein repeat underrepresentation can be a hallmark of method robustness and supports the lack of such artefacts, at least manifested at protein-level. Additionally, it might suggest previously undescribed selection pattern yet to be understood. Fragments of LINEs co-localise with ATP-synt_ab_N ATP synthases (PF02874) and Metallophos phosphoesterases (PF00149). Non-autonomous LTR retrotransposons are preferentially associated with genes coding for Aconitase (PF00330), Catalase (PF00199), Peptidase_M41 (PF01434) and Chitin_synth_1 synthase (PF01644). Remnants of DNA TE are found with genes coding for Glyco_hydro_3_C hydrolase (PF01915) and PNP_UDP_1 phosphorylases (PF01048). Helitron remainings can be found in proteins with Peptidase_S8 (PF00082) and Pkinase (PF00069) domains.

#### TEs with a coding region

Functional transposable elements rarely insert into genes and do not show a statistically significant preference for specific protein domains. Usually, they cluster with other TEs in genomic areas containing fewer genes. Eventually, genes infested by them often carry TE-related domains, and are likely to be TEs misannotated as genes and included into proteomes. TEs tend to insert into other TEs leading to the formation of TE-clusters or composite elements^55,56^.

#### TE location, abundance and hosts’ ecology

**Figure 3:**
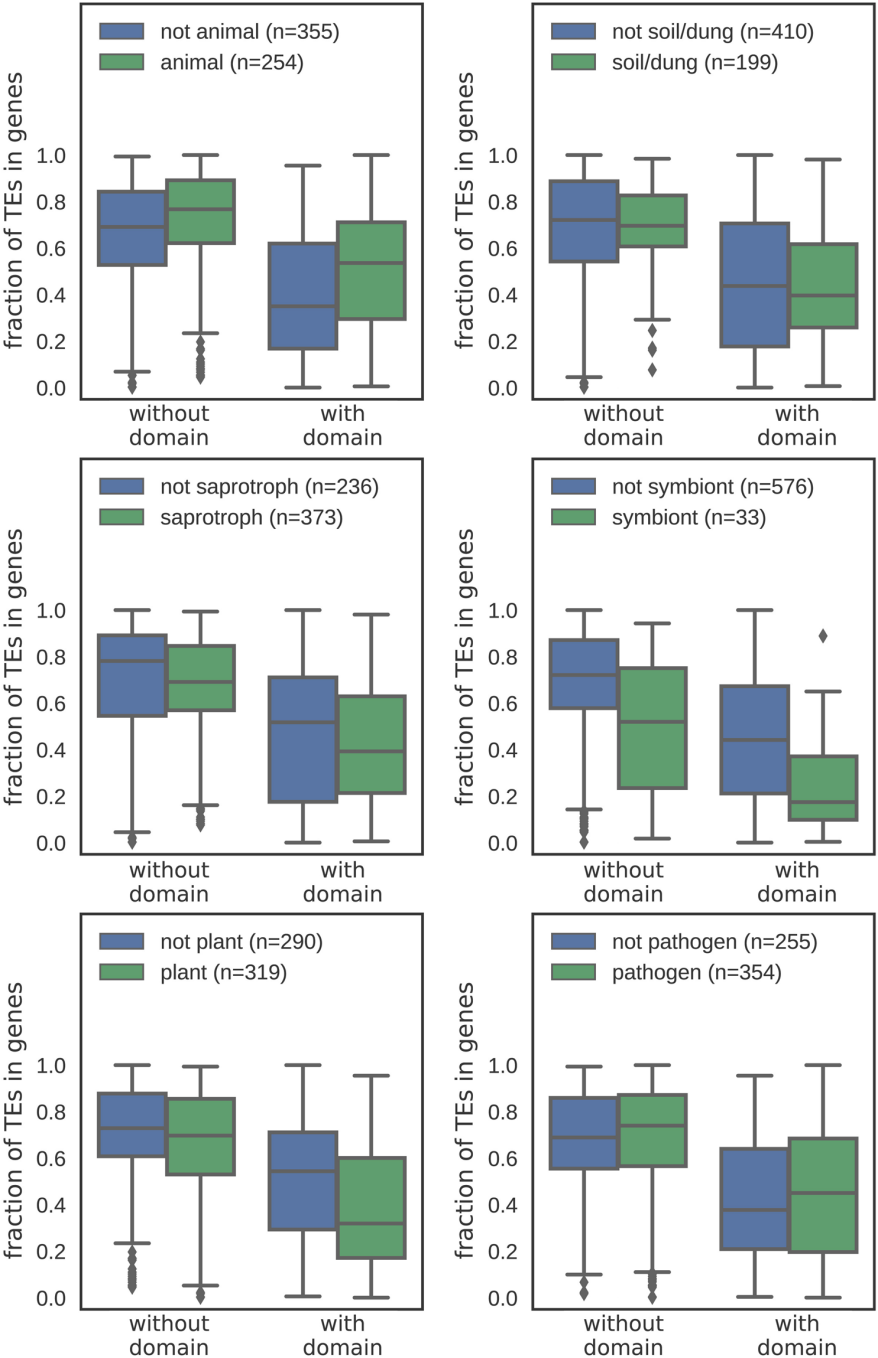
Distribution of TEs in fungi with a given lifestyle. Significance of differences is assessed with Mann–Whitney U test.

Animal-related and pathogenic fungi have more TEs inserted into genes as compared to fungi with other lifestyles (Fig. 3). Plant-related, saprotrophic organisms and those living in soil or on dung have fewer TEs overlapping with genic regions. This effect is straightforwardly correlated with genome compactness of animal pathogenic fungi and genome expansion present in many plant-associated fungi^7^. Genome architecture seems to be the dominant factor determining the relationship between the coding and non-coding genome. Plant-associated fungi have a greater average distance between TEs and genes (370 bp) and fewer genes close to TEs as compared to non-plant related fungi (351 bp between gene and TE on average, p-value = 7.7e-78). Both features are likely attributed to greater genome sizes and overall decrease in gene density.

#### Small secreted proteins

Small secreted proteins (SSPs) are often related with plant-associated lifestyle providing effector activity modulating host performance^57^. Plant-pathogenic fungi are known for their peculiar genome architecture with fast-evolving genomic regions rich in repeat proteins, SSPs and TEs^7^. We tested whether SSPs would indeed cluster with TEs in terms of the neighbourhood defined in this paper. The SSPs were defined either as shorter than 300 amino acids and predicted to be secreted or additionally possessing more than 5% of cysteines (which narrowed the gene list). Regardless of the applied definition, we found no statistical support for the association between SSP neighbourhood with TEs. One of the possible explanations here would be that in fast-evolving genome parts, the maximum distance allowing for TE-gene influence might be bigger than 1 kb averaged for many genomes, with a majority of them having a more uniform genome architecture. Our analyses are also limited by assembly fragmentation particularly affecting repeat-rich genome regions. SSPs understood as genes coding short secreted proteins constituted 3.6% of all neighbouring genes and 6.1% of all protein coding genes. These values varied among genomes with Agaricomycetes (n = 72, mean of 78) having more SSPs in TE neighbourhood than Tremellomycetes (n = 31, mean of 10). Among Pezizomycotina, Eurotiomycetes had less SSPs co-localising with TEs (n = 122, mean 39) than Dothideomycetes (n = 39, mean 69) and Leotiomycetes (n = 33, mean 103), being the most SSP rich in proximity of TEs.

## Discussion

The aim of this study was to explore neighbourhood of fungal transposable elements, either functional or not. TEs are intrinsically linked to genome evolution and constitute minor but still ubiquitous fraction of most fungal genomes. Their roles as potent regulatory elements, genomic parasites and nearly neutral sequences are being revised constantly^58^. According to Arkhipova and others, the most transposable elements remain silent, evolve in a neutral fashion and only a minor fraction gets ever involved in adaptive roles^59^. Our results seem to confirm this perspective, showing no correlation between TE neighbourhood and gene function for many TE families and remnant elements. We might not be able to detect rare events on this large scale e.g. a new regulatory network that uses TEs as TF binding sites. With the advancement of single cell sequencing technologies, it will soon become feasible to observe TE movements and distribution across fungal populations without being limited to model organisms only.

Observed localisation of TEs in 625 fungal genomes shows a dichotomy between relatively young elements depleted in genes, and remnant sequences clearly derived from transposable elements, now more deteriorated, which are equally likely to be found both, within genes and in other locations. This phenomenon provides a pathway to exaptation of TEs, producing new coding regions and utilization as evolutionary raw material for selection. The significance of exaptation in the course of Metazoa evolution has been noted by Scharder and Schmitz in their review on TEs in adaptive evolution^58^.

There are numerous factors shaping TEs distribution ranging from target site preferences in some retrotransposons favouring insertion upstream of polymerase III transcribed genes^60^, strand preference in LTR retrotransposons, via genome rearrangements, to forces of selection and genetic drift acting at a population scale removing TEs with deleterious phenotypes^56,61^. Regardless of insertional preference, present in some TE types, the overall pattern of genomic distribution of both functional and dead elements corroborates a random fashion of TE dispersal within genomes. These genomic parasites remain active outside of genes, where they are less likely to cause deleterious mutations. TEs with a coding region are predominantly in a distance from host genes, what might be related to the repressive effect of many TEs on neighbouring genes^31^. Remnant TEs are not subjected to such constraints and can now be used as raw material for new coding sequences.

The proportion of the genome originated from TEs varies in different fungal lineages as shown previously^7,47^. The bigger the genome, with greater distances between genes, the fewer TEs overlap with genes. The observed pattern suggests the presence of constraints imposed on the size of small genomes, despite multiplication of TEs and randomness of the insertion process. In consequence, small genomes remain small, and large ones grow. The growth of big fungal genomes can be acknowledged to genetic drift, they change with time gaining new slightly deleterious mutations, mobile elements and introns^62–64^. In contrary, the very compact genomes of yeast-like organisms are likely a result of selection^62,65^.

Genome architecture seems to depend on fungus ecology. Most fungi with complex genomes shaped by numerous TEs are plant-associated which has been noticed previously^7,47^. Plant-related fungi are known to use SSPs to deal with plant’s immune reaction. It has been claimed that SSP-coding genes co-localise with TEs, however, we did not observe this effect. The latter effect can be masked by the underrepresentation and fragmentation of repeat rich genomic locations in assemblies. Surprisingly, our findings point at several previously unreported correlations between occurrence of TE-gene overlapping and animal-related and/or pathogenic host lifestyle. It remains an open question whether there is a causative relation between fungal ecology and TEs distribution in the genome – it may be validated by experiments involving multiple high-quality genomes and transcriptomes from closely related taxa differing in lifestyle.

Analysis of an extensive dataset of genomes covering organisms of diverse genome sizes, lifestyles, taxonomic position and TE abundance enabled us to ask whether TE insertions are linked to specific functional categories described previously, such as stress response^66^, mutualism^67^ or phosphorylation^31^. We found no general relationship between the aforementioned biological functions and TE neighbourhood. This finding may suggest that these phenomena are taxon-specific. However, we did find associations between TEs and several unrelated enzyme classes, for particular fungal lineages and TEs classes. Our conclusion supports Arkhipova’s hypothesis that adaptive roles of TEs will remain statistically undetectable and will remain a case-by-case phenomenon. We might hypothesise that TEs can play diverse roles, including adaptive ones, in the course of evolution of particular fungal populations, each being shaped by its constraints. When analysed together, these specific cases are masked by the dominant random and neutral fashion of TE evolution.

## Supplementary information

Supplementary Figures and Legends

Supplementary File S1

Supplementary Tables S1 S2 and S3

## Data Availability

Information processed in statistical analyses is available as Python code and Excel tables.
