# Supplementary material for: Transposable elements contribute to fungal genes and impact fungal lifestyle
Source: Sci Rep. 2019 Mar 13;9:4307. doi: 10.1038/s41598-019-40965-0 (PMC6416283; doi:10.1038/s41598-019-40965-0)
Supplement: Supplementary file 1 — Supplementary Figures and Legends [file 41598_2019_40965_MOESM1_ESM.pdf]

Transposable elements contribute to fungal genes and impact fungal lifestyle

Anna Muszewska[1,*#], Kamil Steczkiewicz[2,#], Marta Stepniewska-Dziubinska[1], Krzysztof Ginalski[2]

[1] Institute of Biochemistry and Biophysics, Polish Academy of Sciences, Pawinskiego 5A, 02-106 Warsaw, Poland

[2] Laboratory of Bioinformatics and Systems Biology, CeNT, University of Warsaw, Zwirki i Wigury 93, 02-089 Warsaw, Poland

Table of contents

Supplementary Figure S1. 3
Supplementary Figure S2. 4
Supplementary Figure S3. 5
Supplementary Table S1. 6
Supplementary Table S2 6
Supplementary Table S3 6
Supplementary File S1. 6

Supplementary Figure S1. Schematic representation of the key steps of the analysis.

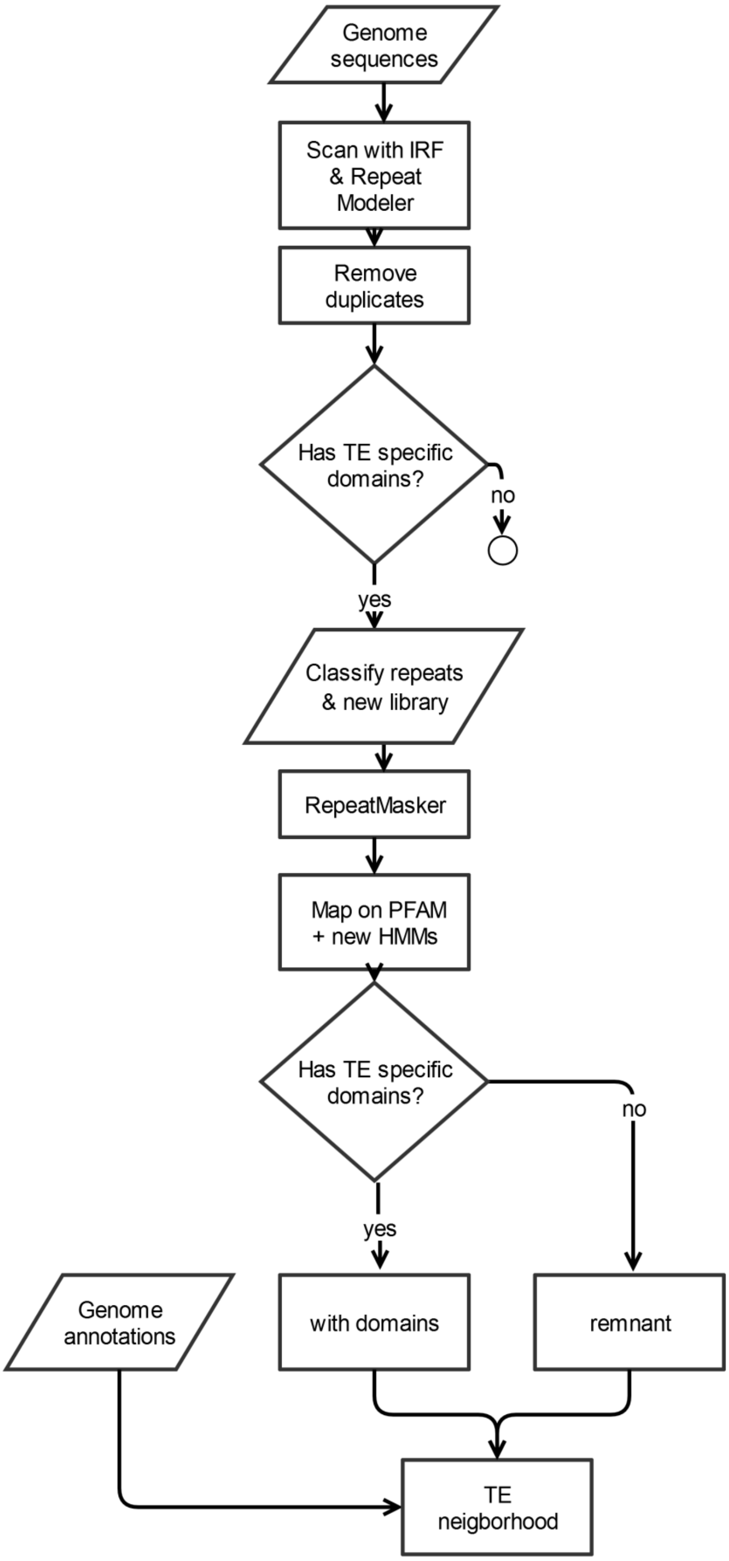

Supplementary Figure S2. Neighbourhood assessment at TE-gene border. (A) If gene encodes no specific TE-related protein domain and overlaps with detected TE, it becomes shortened in favour of TE. (B) Otherwise, when the gene has a TE-related protein domain, TE is expanded to include the gene. (C) If the gene contains an inner TE element (labelled 2), it is this element to be annotated as a neighbour of the analyzed TE (labelled 1).

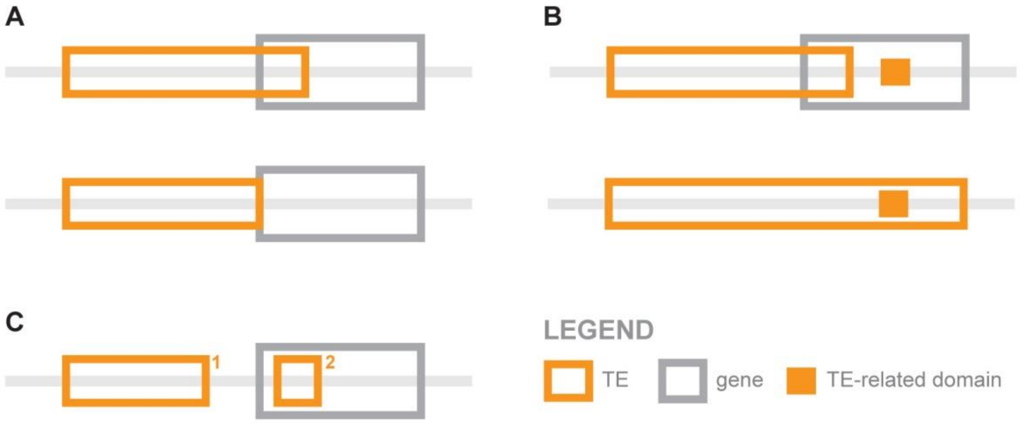

Supplementary Figure S3. Distributions of frequencies of active TEs colocalizing with genes for different fungal subphyla.

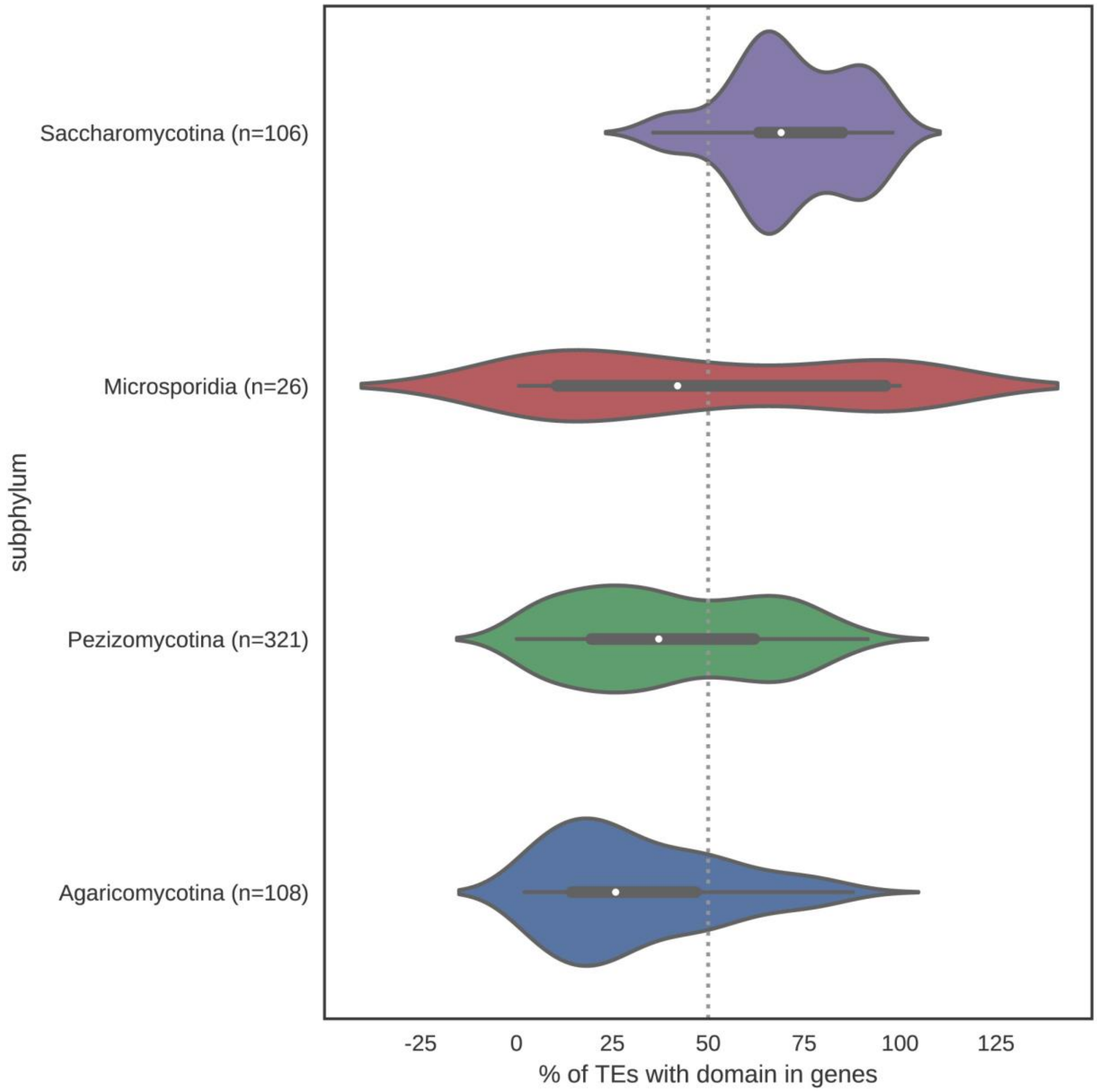

Supplementary Table S1. Assembly list with annotations (xls).

Supplementary Table S2. Protein domains either associated with transposon activity or related to transposons (xls).

Supplementary Table S3. List of protein domains enriched and depleted close to transposons (xls).

Supplementary File S1. Python code in Jupyter notebook (html).
