# Supplementary material for: Transposable elements contribute to fungal genes and impact fungal lifestyle
Source: Sci Rep. 2019 Mar 13;9:4307. doi: 10.1038/s41598-019-40965-0 (PMC6416283; doi:10.1038/s41598-019-40965-0)
Supplement: Supplementary file 2 — Supplementary File S1 [file 41598_2019_40965_MOESM2_ESM.zip › Supplementary File S1.html]

supplementary\_file


In [1]:

```
import warnings
warnings.simplefilter('ignore')

import math
import numpy as np
import scipy
import pandas as pd
import statsmodels.api as sm

import matplotlib.pyplot as plt
import seaborn as sns
sns.set_style('white')
sns.set_color_codes()

%matplotlib inline
```

In [2]:

```
data_path = '../../data/'
```

In [3]:

```
excel_writer = pd.ExcelWriter('supplementary_file.xls')
```

# Helper functions¶

In [4]:

```
phylum_colors = {
    'Ascomycota': (0.65, 0.80, 0.9),
    'Basidiomycota': (1.00, 0.85, 0.50),
    'Blastocladiomycota': (0.70, 0.10, 0.40),
    'Chytridiomycota': (0.15, 0.45, 0.15),
    'Cryptomycota': (0.90, 0.100, 0.10),
    'Microsporidia': (1.0, 0.50, 0.0),
    'Mucoromycota': (0.55, 0.70, 0.40),
    'Zoopagomycota': (0.15, 0.50, 0.70),
    'Neocallimastigomycota': (0.3, 0.3, 0.3)
}


def color_phylum(x, y, table, ax=None):
    """Depict relationship between two variables as scatterplot. Points will be coloured by phylum and
    and figure will be annotated with Pearson's R for the two variables
    
    Args:
        x, y (str): columns names
        table (pd.DataFrame): table containing columns `x`, `y` and 'phylum'
        ax (matplotlib.axes.Axes, optional): axes to use for drawing. If not specified, new axes will be created

    Returns:
        matplotlib.Figure, matplotlib.axes: figure and axes with the scatterplot
    """

    if ax is None:
        fig, ax = plt.subplots(figsize=(7, 7))
    else:
        fig = ax.get_figure()

    for phylum, subtable in table.groupby('phylum'):
        ax.scatter(subtable[x], subtable[y], color=phylum_colors[phylum], edgecolors=(.7, .7, .7),
                   label=phylum)

    
    return fig, ax

def log_binomial_coeff(n, k):
    #use multiplicative formula and calculate logarithms on the fly
    n = float(n)
    w = 0.0

    if k > n/2:    # shorter loop
        k = int(n - k)

    for i in range(1, k + 1):
        w += math.log((n - i + 1.0) / float(i))

    return w


def pval_upperbound(k, n, p):
    """Calculate p-value upperbound for binomial test with k successes, n tries and probability of
    success equal to p
    """
    return np.exp(-2 * (n*p - k)**2 / n)


def enrichment_score(k, n, p, tol=1e-200):
    """Calculate logarithm of probability that given term was found k times in n tries by chance.
    Use binomial distribution:

    log(P) = log((N  k)  * p**k * (1-p)**(N-k)) = log(N k) + k*log(p) + (N-k)*log(1-p)
    """
    
    n = int(n)
    k = int(k)

    log_p = (
        log_binomial_coeff(n, k)
        + k*math.log(p+tol)
        + (n-k)*math.log(1.0-p+tol)
    )

    return log_p


def fill_count_table(count_tab):
    """Fill the table for enrichment analysis.
    
    Args:
        count_tab (pd.DataFrame): table containing the following columns:
            'counts': observed number of successes
            'p': success probability
            'prot_num': number of trials
    """

    count_tab['counts'] = count_tab['counts'].astype('int')
    count_tab['observed_freq'] = count_tab['counts'] / count_tab['prot_num']
    count_tab['expected_counts'] = (count_tab['p'] * count_tab['prot_num']).round().astype(int)
    count_tab['score'] = count_tab.apply(lambda x: enrichment_score(x['counts'], x['prot_num'],
                                                             x['p']), axis=1)
    count_tab['expected_score'] = count_tab.apply(lambda x: enrichment_score(x['expected_counts'],
                                                                                       x['prot_num'],
                                                             x['p']), axis=1)
    count_tab['fold_change'] = count_tab['observed_freq'] / count_tab['p']
    count_tab['fold_change'] = count_tab['fold_change'].apply(lambda x: -1/x if 0 < x < 1 else x)
    count_tab['fold_change'] = count_tab['fold_change'].apply(lambda x: float('-inf') if x == 0 else x)
    count_tab['pval_binom_test'] = pval_upperbound(count_tab['counts'], count_tab['prot_num'], count_tab['p'])
```

# Data¶

## Column names¶

In [5]:

```
assembly_params = ['assembly_len', 'GC', 'intron_per_gene', 'gene_dist', 'total_gene_len', 'num_genes']

taxonomy = ['phylum', 'subphylum', 'class', 'order', 'family', 'genus', 'species', 'species_taxid',
            'organism_name', 'taxid']

host = ['animal', 'fungus', 'plant']
interaction_type = ['pathogen', 'saprotroph', 'symbiont']
environment = ['soil/dung', 'water']
lifestyle = host + interaction_type + environment
```

## Detected TEs and their neighbours¶

In [7]:

```
neighbours = pd.read_csv('%s/neighbours_cleaned.csv' % data_path)
for category in ['L_type', 'L_localization', 'R_type', 'R_localization', 'TE_to_gene', 'localization',
                 'T1', 'T2', 'T3', 'L_targetp_score', 'R_targetp_score', 'targetp_score']:
    neighbours[category] = neighbours[category].astype('category')

neighbours.dtypes
```

Out[7]:

```
assembly_ID          object
contig_ID            object
TE_start              int64
TE_end                int64
L_type             category
L_ID                 object
L_localization     category
L_start               int64
L_end                 int64
R_type             category
R_ID                 object
R_localization     category
R_start               int64
R_end                 int64
TE_to_gene         category
ID                   object
localization       category
TE                   object
domain                 bool
T1                 category
T2                 category
T3                 category
L_targetp_score    category
R_targetp_score    category
targetp_score      category
dtype: object
```

In [8]:

```
neighbours.iloc[10]
```

Out[8]:

```
assembly_ID        GCA_000001985.1
contig_ID               DS995903.1
TE_start                     41872
TE_end                       42214
L_type                        gene
L_ID                    EEA21220.1
L_localization               Other
L_start                      40668
L_end                        48708
R_type                        gene
R_ID                    EEA21220.1
R_localization               Other
R_start                      40668
R_end                        48708
TE_to_gene                  InGene
ID                      EEA21220.1
localization                 Other
TE                       LTR/GYPSY
domain                       False
T1                             LTR
T2                           GYPSY
T3                             NaN
L_targetp_score                  2
R_targetp_score                  2
targetp_score                    2
Name: 10, dtype: object
```

In [9]:

```
num_total = len(neighbours)
print(len(neighbours['assembly_ID'].unique()), 'genomes in the dataset')
print(num_total, 'TEs in total')
num_active = neighbours['domain'].sum()
print(num_active, 'active and', num_total - num_active, 'remntants')
```

```
625 genomes in the dataset
2317558 TEs in total
293746 active and 2023812 remntants
```

## Assembly parameters and lifestyle data¶

In [10]:

```
fungi_data = pd.read_csv('%s/fungi_data_cleaned.csv' % data_path).query('assembly_ID not in @blacklist')
fungi_data.dtypes
```

Out[10]:

```
assembly_ID         object
num_genes            int64
total_gene_len       int64
assembly_len       float64
GC                 float64
intron_per_gene    float64
gene_dist          float64
phylum              object
subphylum           object
class               object
order               object
family              object
genus               object
species             object
species_taxid      float64
organism_name       object
taxid              float64
pathogen           float64
saprotroph         float64
symbiont           float64
animal             float64
fungus             float64
plant              float64
soil/dung          float64
water              float64
dtype: object
```

In [11]:

```
print(len(fungi_data), 'genomes in the dataset')
```

```
625 genomes in the dataset
```

In [12]:

```
fungi_data.iloc[10]
```

Out[12]:

```
assembly_ID           GCA_001023065.1
num_genes                       15226
total_gene_len               23252323
assembly_len              4.34998e+07
GC                              48.76
intron_per_gene                  1.74
gene_dist                     1114.14
phylum                     Ascomycota
subphylum              Pezizomycotina
class                 Sordariomycetes
order                     Hypocreales
family                    Nectriaceae
genus                        Fusarium
species            Fusarium fujikuroi
species_taxid                    5127
organism_name      Fusarium fujikuroi
taxid                            5127
pathogen                            1
saprotroph                          1
symbiont                            0
animal                              0
fungus                              0
plant                               1
soil/dung                           0
water                               0
Name: 10, dtype: object
```

## Protein annotations¶

In [13]:

```
prot_info = pd.read_csv('%s/protein_info.csv' % data_path)
prot_info.head()
```

Out[13]:

|  | ID | aas\_num | assembly\_ID | cys\_num | secreted | ssp |
| --- | --- | --- | --- | --- | --- | --- |
| 0 | EED11412.1 | 1525 | GCA\_000003125.1 | 11 | False | False |
| 1 | EED11413.1 | 1513 | GCA\_000003125.1 | 32 | False | False |
| 2 | EED11414.1 | 249 | GCA\_000003125.1 | 2 | True | False |
| 3 | EED11415.1 | 212 | GCA\_000003125.1 | 4 | False | False |
| 4 | EED11416.1 | 963 | GCA\_000003125.1 | 8 | False | False |

In [14]:

```
pfam = pd.read_csv('%s/pfam.csv' % data_path)
pfam.head()
```

Out[14]:

|  | ID | pfam\_ID | pfam\_name | clan\_ID |
| --- | --- | --- | --- | --- |
| 0 | EEA18456.1 | PF12013 | DUF3505 | PF12013 |
| 1 | EEA18457.1 | PF12013 | DUF3505 | PF12013 |
| 2 | EEA18458.1 | PF12013 | DUF3505 | PF12013 |
| 3 | EEA18461.1 | PF12013 | DUF3505 | PF12013 |
| 4 | EEA18464.1 | PF12013 | DUF3505 | PF12013 |

In [15]:

```
go = pd.read_csv('%s/go.csv' % data_path)
go.head()
```

Out[15]:

|  | pfam\_ID | pfam\_name | GO\_term | GO\_num | ID | clan\_ID |
| --- | --- | --- | --- | --- | --- | --- |
| 0 | PF00001 | 7tm\_1 | G-protein coupled receptor activity | GO:0004930 | KIX02055.1 | CL0192 |
| 1 | PF00001 | 7tm\_1 | G-protein coupled receptor activity | GO:0004930 | KIR76542.1 | CL0192 |
| 2 | PF00001 | 7tm\_1 | G-protein coupled receptor activity | GO:0004930 | KIR83970.1 | CL0192 |
| 3 | PF00001 | 7tm\_1 | G-protein coupled receptor activity | GO:0004930 | KIR91110.1 | CL0192 |
| 4 | PF00001 | 7tm\_1 | G-protein coupled receptor activity | GO:0004930 | KIR96427.1 | CL0192 |

In [16]:

```
TE_domains = pd.read_csv('%s/transpo_domains_2018.tab' % data_path, sep='\t', header=None).dropna()
TE_domains.columns = ['TE', 'database', 'domain', 'domain_id']
TE_domains = TE_domains[TE_domains['database'] != 'database']

TE_domains.head()
```

Out[16]:

|  | TE | database | domain | domain\_id |
| --- | --- | --- | --- | --- |
| 0 | DNA/Academ | cdd | recQ\_fam | 129701 |
| 1 | DNA/Academ | cdd | HELICc | 197757 |
| 2 | DNA/Academ | cdd | DEXDc | 214692 |
| 3 | DNA/Academ | cdd | RecQ | 223588 |
| 4 | DNA/Academ | cdd | DEXDc | 238005 |

In [17]:

```
# set of TE-specific domains to filterout from enrichment analysis

domains_filter = {'T1': {}, 'T2': {}}

for _, row in TE_domains.iterrows():
    # skip cdd info - we only have pfam IDs
    if row['database'] == 'cdd':
        continue

    te = row['TE'].split('/')
    if len(te) == 1:
        t1 = te[0].upper()
        t2 = None
    else:
        t1, t2 = te
        t1 = t1.upper()
        t2 = t2.split('-')[0].upper()
    if t1 not in domains_filter['T1']:
        domains_filter['T1'][t1] = set()
    domains_filter['T1'][t1].add(row['domain_id'])
    if t2 is not None:
        if t2 not in domains_filter['T2']:
            domains_filter['T2'][t2] = set()
        domains_filter['T2'][t2].add(row['domain_id'])
```

# Basic statistics¶

## Neighbour type¶

In [18]:

```
size = len(neighbours)
```

In [19]:

```
relationships = {

    'in_gene': (~neighbours['ID'].isnull()),

    'L=null, R=null': (neighbours['ID'].isnull()
                       & neighbours['L_type'].isnull()
                       & neighbours['R_type'].isnull()),

    'L=null, R=TE': (neighbours['ID'].isnull()
                     & neighbours['L_type'].isnull()
                     & (neighbours['R_type'] == 'te')),

    'L=TE,   R=null': (neighbours['ID'].isnull()
                       & neighbours['R_type'].isnull()
                       & (neighbours['L_type'] == 'te')),

    'L=null, R=gene': (neighbours['ID'].isnull()
                       & neighbours['L_type'].isnull()
                       & (neighbours['R_type'] == 'gene')),

    'L=gene, R=null': (neighbours['ID'].isnull()
                       & neighbours['R_type'].isnull()
                       & (neighbours['L_type'] == 'gene')),

    'L=TE,   R=TE': (neighbours['ID'].isnull()
                     & (neighbours['L_type'] == 'te')
                     & (neighbours['R_type'] == 'te')),

    'L=TE,   R=gene': (neighbours['ID'].isnull()
                       & (neighbours['L_type'] == 'te')
                       & (neighbours['R_type'] == 'gene')),

    'L=gene, R=TE': (neighbours['ID'].isnull()
                     & (neighbours['L_type'] == 'gene')
                     & (neighbours['R_type'] == 'te')),

    'L=gene, R=gene': (neighbours['ID'].isnull()
                       & (neighbours['L_type'] == 'gene')
                       & (neighbours['R_type'] == 'gene')),

}
```

In [20]:

```
relationship_types = list(relationships)
```

In [21]:

```
# make sure that defined neighbourhoods are exclusive
for i, r1 in enumerate(relationship_types):
    for r2 in relationship_types[:i]:
        assert (relationships[r1] & relationships[r2]).sum() == 0, '%s & %s' % (r1, r2)
```

### For all TEs¶

In [22]:

```
df = pd.DataFrame(columns=['neighbourhood', 'count'])
i = 0

for r, idx in relationships.items():
    print('%-14s  %7d (%5.2f%%)' % (r, idx.sum(), (100 * idx.sum() / size).round(2)))
    df.loc[i] = [r, idx.sum()]
    i += 1
```

```
in_gene         1071707 (46.24%)
L=null, R=null   247528 (10.68%)
L=null, R=TE     188308 ( 8.13%)
L=TE,   R=null   189562 ( 8.18%)
L=null, R=gene    78593 ( 3.39%)
L=gene, R=null    79543 ( 3.43%)
L=TE,   R=TE     300526 (12.97%)
L=TE,   R=gene    58878 ( 2.54%)
L=gene, R=TE      60153 ( 2.60%)
L=gene, R=gene    42760 ( 1.85%)
```

## For TEs with/without TE-related domains separately¶

In [23]:

```
with_domain = neighbours['domain']

for wks_name, query in (('TE_domain_neighbourhood', with_domain), ('TE_no_domain_neighbourhood', ~with_domain)):

    print(wks_name)
    df = pd.DataFrame(columns=['neighbourhood', 'count'])
    i = 0

    for r, idx in relationships.items():
        idx &= query
        print('%-14s  %7d (%5.2f%%)' % (r, idx.sum(), (100 * idx.sum() / query.sum()).round(2)))
        df.loc[i] = [r, idx.sum()]
        i += 1

    df.to_excel(excel_writer, sheet_name=wks_name)
    print()

excel_writer.save()
```

```
TE_domain_neighbourhood
in_gene           46789 (15.93%)
L=null, R=null    54080 (18.41%)
L=null, R=TE      41194 (14.02%)
L=TE,   R=null    41688 (14.19%)
L=null, R=gene    11871 ( 4.04%)
L=gene, R=null    11717 ( 3.99%)
L=TE,   R=TE      62502 (21.28%)
L=TE,   R=gene     8878 ( 3.02%)
L=gene, R=TE       9108 ( 3.10%)
L=gene, R=gene     5919 ( 2.02%)

TE_no_domain_neighbourhood
in_gene         1024918 (50.64%)
L=null, R=null   193448 ( 9.56%)
L=null, R=TE     147114 ( 7.27%)
L=TE,   R=null   147874 ( 7.31%)
L=null, R=gene    66722 ( 3.30%)
L=gene, R=null    67826 ( 3.35%)
L=TE,   R=TE     238024 (11.76%)
L=TE,   R=gene    50000 ( 2.47%)
L=gene, R=TE      51045 ( 2.52%)
L=gene, R=gene    36841 ( 1.82%)
```

## Groupped by TE type¶

In [24]:

```
# get TE types with at least 1000 occurences of active TE

frequent_TEs = list(
    neighbours
    .query('domain')
    ['T2']
    .value_counts()
    .loc[lambda x: x > 1000]
    .index
)
frequent_TEs
```

Out[24]:

```
['GYPSY',
 'COPIA',
 'TCMAR',
 'TAD1',
 'HAT',
 'HELITRON',
 'ZISUPTON',
 'PIF',
 'MULE',
 'DIRS',
 'CMC',
 'L1',
 'ACADEM',
 'PIGGYBAC',
 'GINGER',
 'MERLIN']
```

In [25]:

```
for wks_name, query in (('TE_domain_in_gene', 'T2 in @frequent_TEs and domain'),
                       ('TE_no_domain_in_gene', 'T2 in @frequent_TEs and not domain')):

    print(wks_name)

    df = pd.DataFrame(columns=['T1', 'T2', 'all', 'in_gene', 'L_is_gene', 'R_is_gene'])
    i = 0
    for (T1, T2), tab in neighbours.query(query).groupby(['T1', 'T2']):
        all_TEs = len(tab)
        in_gene = (~tab['ID'].isnull()).sum()
        L_is_gene = (tab[tab['ID'].isnull()]['L_type'] == 'gene').sum()
        R_is_gene = (tab[tab['ID'].isnull()]['R_type'] == 'gene').sum()
        print('%5s %-12s\tin_gene=%6d (%5.2f%%) \tL=%6d; R=%6d' % (
            T1,
            T2,
            in_gene,
            100 * in_gene / all_TEs,
            L_is_gene,
            R_is_gene))
        df.loc[i] = [T1, T2, all_TEs, in_gene, L_is_gene, R_is_gene]
        i += 1

    df.to_excel(excel_writer, sheet_name=wks_name)
    print()

excel_writer.save()
```

```
TE_domain_in_gene
  DNA ACADEM      	in_gene=  1568 (80.91%) 	L=    56; R=    53
  DNA CMC         	in_gene=   567 (18.23%) 	L=   397; R=   410
  DNA GINGER      	in_gene=    51 ( 2.93%) 	L=   216; R=   214
  DNA HAT         	in_gene=   426 ( 3.84%) 	L=  1732; R=  1659
  DNA MERLIN      	in_gene=     9 ( 0.67%) 	L=   176; R=   161
  DNA MULE        	in_gene=   254 ( 5.94%) 	L=   775; R=   773
  DNA PIF         	in_gene=   232 ( 4.92%) 	L=   854; R=   837
  DNA PIGGYBAC    	in_gene=    21 ( 1.17%) 	L=   359; R=   360
  DNA TCMAR       	in_gene=  1135 ( 3.79%) 	L=  6495; R=  6385
  DNA ZISUPTON    	in_gene=  3820 (63.63%) 	L=   328; R=   300
 LINE L1          	in_gene=   512 (23.80%) 	L=   268; R=   282
 LINE TAD1        	in_gene=   332 ( 1.60%) 	L=  1469; R=  1483
  LTR COPIA       	in_gene=  1316 ( 3.43%) 	L=  3156; R=  3262
  LTR DIRS        	in_gene=   196 ( 5.50%) 	L=   289; R=   272
  LTR GYPSY       	in_gene=  3969 ( 4.67%) 	L=  4659; R=  4410
   RC HELITRON    	in_gene=  1501 (17.32%) 	L=  1010; R=  1115

TE_no_domain_in_gene
  DNA ACADEM      	in_gene=   467 (28.55%) 	L=   249; R=   258
  DNA CMC         	in_gene= 28254 (69.54%) 	L=  2296; R=  2199
  DNA GINGER      	in_gene=  2336 (39.16%) 	L=   621; R=   630
  DNA HAT         	in_gene= 36492 (39.18%) 	L=  9358; R=  9200
  DNA MERLIN      	in_gene=  1966 (22.18%) 	L=  1100; R=  1121
  DNA MULE        	in_gene= 23703 (43.82%) 	L=  5801; R=  5718
  DNA PIF         	in_gene= 13777 (34.09%) 	L=  4647; R=  4573
  DNA PIGGYBAC    	in_gene= 12013 (62.92%) 	L=  1630; R=  1643
  DNA TCMAR       	in_gene=365093 (67.28%) 	L= 37941; R= 37052
  DNA ZISUPTON    	in_gene=  2918 (88.56%) 	L=    62; R=    48
 LINE L1          	in_gene= 14553 (68.43%) 	L=  1537; R=  1488
 LINE TAD1        	in_gene= 59499 (40.18%) 	L=  9675; R=  9443
  LTR COPIA       	in_gene= 90768 (41.71%) 	L= 18400; R= 18098
  LTR DIRS        	in_gene=  8472 (51.32%) 	L=  1462; R=  1494
  LTR GYPSY       	in_gene=164431 (36.52%) 	L= 34631; R= 34467
   RC HELITRON    	in_gene= 23923 (50.48%) 	L=  4454; R=  4545
```

# TEs aboundance vs genome properties¶

In [26]:

```
te_lengths = neighbours.groupby(['assembly_ID', 'domain']).size()
te_lengths.name = 'num_TEs'
te_lengths = te_lengths.reset_index()

te_lengths.head()
```

Out[26]:

|  | assembly\_ID | domain | num\_TEs |
| --- | --- | --- | --- |
| 0 | GCA\_000001985.1 | False | 2481 |
| 1 | GCA\_000001985.1 | True | 317 |
| 2 | GCA\_000002495.2 | False | 2682 |
| 3 | GCA\_000002495.2 | True | 865 |
| 4 | GCA\_000002515.1 | False | 886 |

In [27]:

```
te_fraction = (
    neighbours
    .groupby(['assembly_ID', 'domain'])
    .agg({'ID': lambda x: (~x.isnull()).sum()})
    .reset_index()
    .rename(columns={'ID': 'TEs_in_gene'})
)

te_fraction = pd.merge(pd.merge(te_lengths, fungi_data), te_fraction)
te_fraction['TEs_in_gene_fraction'] = te_fraction['TEs_in_gene'] / te_fraction['num_TEs']
te_fraction['genes_in_genome_fraction'] = te_fraction['total_gene_len'] / te_fraction['assembly_len']

te_fraction.iloc[10]
```

Out[27]:

```
assembly_ID                             GCA_000002655.1
domain                                            False
num_TEs                                            2529
num_genes                                          9916
total_gene_len                                 15817596
assembly_len                                 2.9385e+07
GC                                                 49.8
intron_per_gene                                    1.89
gene_dist                                       1358.78
phylum                                       Ascomycota
subphylum                                Pezizomycotina
class                                    Eurotiomycetes
order                                        Eurotiales
family                                   Aspergillaceae
genus                                       Aspergillus
species                           Aspergillus fumigatus
species_taxid                                    746128
organism_name               Aspergillus fumigatus Af293
taxid                                            330879
pathogen                                              0
saprotroph                                            1
symbiont                                              0
animal                                                1
fungus                                                0
plant                                                 1
soil/dung                                             1
water                                                 0
TEs_in_gene                                        2090
TEs_in_gene_fraction                           0.826414
genes_in_genome_fraction                       0.538289
Name: 10, dtype: object
```

In [28]:

```
for figname, query in (('Fig.2.A_TE_domain_in_genes', 'domain'),
                       ('Fig.2.B_TE_no_domain_in_genes', 'not domain')):

    tab = te_fraction.query(query)
    x = sm.add_constant(tab['genes_in_genome_fraction'], prepend=False)
    y = np.array((tab['TEs_in_gene'], (tab['num_TEs'] - tab['TEs_in_gene']))).T
    glm_binom = sm.GLM(y, x, family=sm.families.Binomial())
    res = glm_binom.fit()
    print(res.summary())
    
    fig, ax = color_phylum('genes_in_genome_fraction', 'TEs_in_gene_fraction', tab)
    ax.set_xlabel('fraction of genes in genome')
    ax.set_ylabel('fraction of TEs in genes')
    ymin, ymax = ax.get_ylim()
    ax.set_ylim(ymin, ymax*1.1)
    ax.text(0.9, 0.95, '$R^{2}_{McF}$ = %.2f' % (1 - (res.llf / res.llnull)), ha='center', va='center',
            transform=ax.transAxes)
    ax.legend()

    fig.savefig('%s.pdf' % figname)
```

```
                 Generalized Linear Model Regression Results                  
==============================================================================
Dep. Variable:           ['y1', 'y2']   No. Observations:                  625
Model:                            GLM   Df Residuals:                      623
Model Family:                Binomial   Df Model:                            1
Link Function:                  logit   Scale:                          1.0000
Method:                          IRLS   Log-Likelihood:                -18962.
Date:                Sat, 02 Feb 2019   Deviance:                       34668.
Time:                        14:20:52   Pearson chi2:                 3.87e+04
No. Iterations:                     6   Covariance Type:             nonrobust
============================================================================================
                               coef    std err          z      P>|z|      [0.025      0.975]
--------------------------------------------------------------------------------------------
genes_in_genome_fraction     7.2371      0.043    169.302      0.000       7.153       7.321
const                       -5.1815      0.024   -219.294      0.000      -5.228      -5.135
============================================================================================
                 Generalized Linear Model Regression Results                  
==============================================================================
Dep. Variable:           ['y1', 'y2']   No. Observations:                  625
Model:                            GLM   Df Residuals:                      623
Model Family:                Binomial   Df Model:                            1
Link Function:                  logit   Scale:                          1.0000
Method:                          IRLS   Log-Likelihood:            -1.4937e+05
Date:                Sat, 02 Feb 2019   Deviance:                   2.9402e+05
Time:                        14:20:53   Pearson chi2:                 2.88e+05
No. Iterations:                     5   Covariance Type:             nonrobust
============================================================================================
                               coef    std err          z      P>|z|      [0.025      0.975]
--------------------------------------------------------------------------------------------
genes_in_genome_fraction     7.6036      0.013    601.574      0.000       7.579       7.628
const                       -3.6772      0.007   -562.380      0.000      -3.690      -3.664
============================================================================================
```

In [29]:

```
fig, ax = plt.subplots()

for label, query in (('with domain', 'domain'), ('without domain', 'not domain')):
    sns.distplot(te_fraction.query(query)['TEs_in_gene_fraction'], norm_hist=True, kde=False, label=label, ax=ax)

ax.set_xlabel('% of TEs in genes')
ax.set_ylabel('% of assemblies')
ax.set_xticks(np.arange(0, 1.1, 0.1))
ax.set_xticklabels(range(0, 110, 10))
ax.legend()

fig.savefig('Fig.2.C_TEs_in_genes_distribution.pdf')
```

```
/home/marta/miniconda3/lib/python3.6/site-packages/matplotlib/axes/_axes.py:6462: UserWarning: The 'normed' kwarg is deprecated, and has been replaced by the 'density' kwarg.
  warnings.warn("The 'normed' kwarg is deprecated, and has been "
/home/marta/miniconda3/lib/python3.6/site-packages/matplotlib/axes/_axes.py:6462: UserWarning: The 'normed' kwarg is deprecated, and has been replaced by the 'density' kwarg.
  warnings.warn("The 'normed' kwarg is deprecated, and has been "
```

In [30]:

```
count_subphylum = te_fraction.query('domain').groupby('subphylum').size()
count_subphylum.sort_values(ascending=False)
```

Out[30]:

```
subphylum
Pezizomycotina           321
Agaricomycotina          108
Saccharomycotina         106
Microsporidia             26
Ustilaginomycotina        18
Pucciniomycotina          12
Mucoromycotina            12
Taphrinomycotina          10
Chytridiomycota            4
Mortierellomycotina        2
Glomeromycotina            2
Entomophthoromycotina      1
Cryptomycota               1
Blastocladiomycota         1
dtype: int64
```

In [31]:

```
frequent_subphyla = list(count_subphylum.loc[lambda x: x > 20].index)

subphyla_order = (
    te_fraction
    .query('domain')
    .groupby('subphylum')
    ['TEs_in_gene_fraction']
    .median()
    .loc[frequent_subphyla]
    .sort_values()
    .index
)
```

In [32]:

```
fig, ax = plt.subplots(figsize=(7, 7))
sns.violinplot(y='subphylum', x='TEs_in_gene_fraction', data=te_fraction.query('domain'), order=subphyla_order, orient='h',ax=ax)
ax.set_xlim(-0.5, 1.5)
ax.set_ylim(-0.5, 3.5)
ax.set_xticks(np.arange(-0.25, 1.3, 0.25))
ax.set_xticklabels(range(-25, 130, 25))
ax.set_xlabel('% of TEs with domain in genes')
ax.set_yticklabels(['%15s (n=%s)' % (label.get_text(), count_subphylum.loc[label.get_text()])
                    for label in ax.get_yticklabels()])
ax.vlines(0.5, -0.5, 3.5, linestyles=':', color='gray')
fig.tight_layout()

fig.savefig('Supp_fig_S1.pdf')
```

In [33]:

```
for class_name, last_tax_category in (('Saccharomycetes', 'genus'), ('Microsporidia', 'genus'),
                                      ('Eurotiomycetes', 'order'), ('Sordariomycetes', 'order')):
    display(
        (te_fraction
         [te_fraction['class'] == class_name]
         .query('domain')
         .groupby(['phylum', 'subphylum', 'class', last_tax_category])
         .agg({'TEs_in_gene_fraction': ['min', 'mean', 'median', 'max', len]})
         .round(2))
    )
```

|  |  |  |  | TEs\_in\_gene\_fraction | | | | |
| --- | --- | --- | --- | --- | --- | --- | --- | --- |
|  |  |  |  | min | mean | median | max | len |
| phylum | subphylum | class | genus |  |  |  |  |  |
| Ascomycota | Saccharomycotina | Saccharomycetes | Brettanomyces | 0.43 | 0.43 | 0.43 | 0.43 | 1.0 |
|  |  |  | Candida | 0.61 | 0.67 | 0.66 | 0.88 | 30.0 |
|  |  |  | Clavispora | 0.75 | 0.84 | 0.84 | 0.93 | 2.0 |
|  |  |  | Cyberlindnera | 0.74 | 0.74 | 0.74 | 0.74 | 1.0 |
|  |  |  | Debaryomyces | 0.83 | 0.84 | 0.84 | 0.85 | 2.0 |
|  |  |  | Eremothecium | 0.90 | 0.93 | 0.93 | 0.94 | 3.0 |
|  |  |  | Hanseniaspora | 0.57 | 0.66 | 0.66 | 0.74 | 2.0 |
|  |  |  | Kazachstania | 0.64 | 0.76 | 0.76 | 0.89 | 2.0 |
|  |  |  | Kluyveromyces | 0.60 | 0.79 | 0.81 | 0.95 | 4.0 |
|  |  |  | Komagataella | 0.93 | 0.94 | 0.94 | 0.95 | 2.0 |
|  |  |  | Kuraishia | 0.94 | 0.94 | 0.94 | 0.94 | 1.0 |
|  |  |  | Lachancea | 0.88 | 0.90 | 0.90 | 0.92 | 2.0 |
|  |  |  | Lodderomyces | 0.41 | 0.41 | 0.41 | 0.41 | 1.0 |
|  |  |  | Metschnikowia | 0.58 | 0.58 | 0.58 | 0.58 | 1.0 |
|  |  |  | Meyerozyma | 0.87 | 0.87 | 0.87 | 0.87 | 1.0 |
|  |  |  | Millerozyma | 0.98 | 0.98 | 0.98 | 0.98 | 1.0 |
|  |  |  | Nakaseomyces | 0.89 | 0.92 | 0.93 | 0.94 | 7.0 |
|  |  |  | Naumovozyma | 0.67 | 0.67 | 0.67 | 0.67 | 2.0 |
|  |  |  | Ogataea | 0.83 | 0.83 | 0.83 | 0.84 | 2.0 |
|  |  |  | Pichia | 0.55 | 0.55 | 0.55 | 0.55 | 1.0 |
|  |  |  | Saccharomyces | 0.37 | 0.61 | 0.62 | 0.82 | 25.0 |
|  |  |  | Saccharomycetaceae | 0.85 | 0.85 | 0.85 | 0.85 | 1.0 |
|  |  |  | Scheffersomyces | 0.36 | 0.36 | 0.36 | 0.36 | 1.0 |
|  |  |  | Spathaspora | 0.83 | 0.83 | 0.83 | 0.83 | 1.0 |
|  |  |  | Tetrapisispora | 0.51 | 0.61 | 0.61 | 0.72 | 2.0 |
|  |  |  | Torulaspora | 0.88 | 0.88 | 0.88 | 0.88 | 1.0 |
|  |  |  | Vanderwaltozyma | 0.63 | 0.63 | 0.63 | 0.63 | 1.0 |
|  |  |  | Wickerhamomyces | 0.84 | 0.84 | 0.84 | 0.84 | 1.0 |
|  |  |  | Yamadazyma | 0.75 | 0.75 | 0.75 | 0.75 | 1.0 |
|  |  |  | Yarrowia | 0.55 | 0.55 | 0.55 | 0.55 | 1.0 |
|  |  |  | Zygosaccharomyces | 0.87 | 0.92 | 0.93 | 0.95 | 3.0 |

|  |  |  |  | TEs\_in\_gene\_fraction | | | | |
| --- | --- | --- | --- | --- | --- | --- | --- | --- |
|  |  |  |  | min | mean | median | max | len |
| phylum | subphylum | class | genus |  |  |  |  |  |
| Microsporidia | Microsporidia | Microsporidia | Anncaliia | 0.01 | 0.02 | 0.02 | 0.02 | 2.0 |
|  |  |  | Edhazardia | 0.10 | 0.10 | 0.10 | 0.10 | 1.0 |
|  |  |  | Encephalitozoon | 1.00 | 1.00 | 1.00 | 1.00 | 5.0 |
|  |  |  | Enterocytozoon | 0.21 | 0.21 | 0.21 | 0.21 | 1.0 |
|  |  |  | Mitosporidium | 0.85 | 0.85 | 0.85 | 0.85 | 1.0 |
|  |  |  | Nematocida | 0.41 | 0.53 | 0.50 | 0.82 | 6.0 |
|  |  |  | Nosema | 0.01 | 0.07 | 0.07 | 0.13 | 4.0 |
|  |  |  | Ordospora | 1.00 | 1.00 | 1.00 | 1.00 | 1.0 |
|  |  |  | Pseudoloma | 0.17 | 0.17 | 0.17 | 0.17 | 1.0 |
|  |  |  | Spraguea | 0.30 | 0.30 | 0.30 | 0.30 | 1.0 |
|  |  |  | Trachipleistophora | 0.18 | 0.18 | 0.18 | 0.18 | 1.0 |
|  |  |  | Vavraia | 0.07 | 0.07 | 0.07 | 0.07 | 1.0 |
|  |  |  | Vittaforma | 1.00 | 1.00 | 1.00 | 1.00 | 1.0 |

|  |  |  |  | TEs\_in\_gene\_fraction | | | | |
| --- | --- | --- | --- | --- | --- | --- | --- | --- |
|  |  |  |  | min | mean | median | max | len |
| phylum | subphylum | class | order |  |  |  |  |  |
| Ascomycota | Pezizomycotina | Eurotiomycetes | Chaetothyriales | 0.21 | 0.60 | 0.65 | 0.92 | 26.0 |
|  |  |  | Eurotiales | 0.09 | 0.46 | 0.41 | 0.91 | 49.0 |
|  |  |  | Onygenales | 0.01 | 0.41 | 0.38 | 0.85 | 45.0 |
|  |  |  | Phaeomoniellales | 0.71 | 0.71 | 0.71 | 0.71 | 1.0 |
|  |  |  | Verrucariales | 0.11 | 0.11 | 0.11 | 0.11 | 1.0 |

|  |  |  |  | TEs\_in\_gene\_fraction | | | | |
| --- | --- | --- | --- | --- | --- | --- | --- | --- |
|  |  |  |  | min | mean | median | max | len |
| phylum | subphylum | class | order |  |  |  |  |  |
| Ascomycota | Pezizomycotina | Sordariomycetes | Diaporthales | 0.18 | 0.64 | 0.75 | 0.87 | 4.0 |
|  |  |  | Glomerellales | 0.05 | 0.46 | 0.44 | 0.85 | 17.0 |
|  |  |  | Hypocreales | 0.04 | 0.36 | 0.31 | 0.84 | 71.0 |
|  |  |  | Magnaporthales | 0.08 | 0.36 | 0.45 | 0.63 | 5.0 |
|  |  |  | Microascales | 0.17 | 0.39 | 0.30 | 0.70 | 3.0 |
|  |  |  | Ophiostomatales | 0.21 | 0.49 | 0.59 | 0.66 | 6.0 |
|  |  |  | Sordariales | 0.06 | 0.28 | 0.24 | 0.64 | 11.0 |
|  |  |  | Togniniales | 0.38 | 0.38 | 0.38 | 0.38 | 1.0 |
|  |  |  | Xylariales | 0.37 | 0.59 | 0.59 | 0.82 | 4.0 |

# Types of proteins with TEs inserted¶

In [34]:

```
T2s = [
    'ACADEM',
    'CMC',
    'CRYPTON',
    'HAT',
    'MULE',
    'PIF',
    'TCMAR',
    'ZISUPTON',
    'L1',
    'TAD1',
    'COPIA',
    'DIRS',
    'GYPSY',
    'HELITRON',
]
```

In [35]:

```
T1s = list(neighbours.query('T2 in @T2s')['T1'].unique())
T1s
```

Out[35]:

```
['LTR', 'DNA', 'LINE', 'RC']
```

In [36]:

```
neigh_pfam = pd.merge(
    pd.merge(
        neighbours.query('T1 in @T1s or T2 in @T2s'),
        fungi_data)
    [['domain', 'ID', 'T1', 'T2', 'class']]
    .dropna()
    .drop_duplicates(),
    
    pfam
)
```

## All families related to analyzed fungi¶

In [37]:

```
# number of all annotated proteins in the genomes
all_prot_num = len(pfam['ID'].unique())
all_prot_num
```

Out[37]:

```
4371034
```

In [38]:

```
# number of proteins from each family
all_counts = (
    pfam
    .groupby('pfam_ID')
    .agg({'ID': len, 'pfam_name': lambda x: x.unique()[0]})
    .rename(columns={'ID': 'all_counts'})
    .reset_index()
)

all_counts['p'] = all_counts['all_counts'] / all_prot_num

all_counts.head()
```

Out[38]:

|  | pfam\_ID | all\_counts | pfam\_name | p |
| --- | --- | --- | --- | --- |
| 0 | PF00001 | 313 | 7tm\_1 | 0.000072 |
| 1 | PF00002 | 427 | 7tm\_2 | 0.000098 |
| 2 | PF00003 | 228 | 7tm\_3 | 0.000052 |
| 3 | PF00004 | 25779 | AAA | 0.005898 |
| 4 | PF00005 | 27882 | ABC\_tran | 0.006379 |

## PFAM enrichment¶

In [39]:

```
for level in ('T1', 'T2'):

    # group records by TE type (with/without domain, TE family)
    print(level)
    counts = (
        neigh_pfam
        .query(f'{level} in @{level}s')
        .groupby(['domain', level, 'pfam_ID'])
        .agg({'ID': lambda x: len(x),                  # count proteins from given pfam with TE of given type
              'pfam_name': lambda x: x.unique()[0],    # get pfam name
              'class': lambda x: tuple(x.unique())})   # get taxonomic info
        .rename(columns={'ID': 'counts'})
        .dropna(subset=['counts'])
        .reset_index()
    )

    assert not counts.duplicated().any()

    # get total number of proteins with TE insterted
    prot_nums = counts.groupby(['domain', level])['counts'].sum()

    count_table = pd.merge(
        counts,
        all_counts,
        on=['pfam_ID'],
        how='right',
        suffixes=('s', 's_all')
    ).dropna(subset=[level]).fillna({'counts': 0})

    index = list(zip(count_table['domain'], count_table[level]))
    count_table['prot_num'] = prot_nums.loc[index].values.astype(int)

    fill_count_table(count_table)

    assert (count_table['prot_num'] > 0).all()
    assert not count_table.duplicated().any()
    assert not count_table.isnull().any().any()

    hits = count_table.query('pval_binom_test < 0.05')

    # filter out TE-related proteins
    indices_to_keep = []
    for idx, row in hits.iterrows():
        if row[level] not in domains_filter[level] or row['pfam_ID'] not in domains_filter[level][row[level]]:
            indices_to_keep.append(idx)
    hits = hits.loc[indices_to_keep]

    hits['sort_key'] = hits['fold_change'].abs()
    del hits['pfam_names_all']

    for (domain, te_type), hits_tab in hits.groupby(['domain', level]):
        domain = 'domain' if domain else 'no_domain'
        print(domain, te_type, len(hits_tab))
        if len(hits_tab) == 0:
            continue

        hits_tab['class'] = hits_tab['class'].str.join(', ')
        hits_tab = hits_tab.sort_values(['all_counts', 'sort_key'], ascending=False)

        wks_name = '%s_%s_pfam' % (te_type, domain)
        hits_tab.to_excel(excel_writer, sheet_name=wks_name)
    print()

excel_writer.save()
```

```
T1
no_domain DNA 58
no_domain LINE 48
no_domain LTR 42
no_domain RC 47
domain DNA 9
domain LINE 3
domain LTR 16
domain RC 1

T2
no_domain ACADEM 2
no_domain CMC 39
no_domain COPIA 60
no_domain CRYPTON 33
no_domain DIRS 25
no_domain GYPSY 41
no_domain HAT 34
no_domain HELITRON 47
no_domain L1 45
no_domain MULE 37
no_domain PIF 30
no_domain TAD1 43
no_domain TCMAR 59
no_domain ZISUPTON 12
domain ACADEM 8
domain COPIA 1
domain GYPSY 18
domain HAT 1
domain HELITRON 1
domain L1 6
domain TAD1 3
domain TCMAR 6
domain ZISUPTON 1
```

## GO enrichment¶

In [40]:

```
# number of all proteins with GO annotations
all_go_prot_num = len(go['ID'].unique())
all_go_prot_num
```

Out[40]:

```
2731525
```

In [41]:

```
all_go_counts = (
    go
    .groupby('GO_num')
    .agg({'ID': len, 'GO_term': lambda x: x.unique()[0], 'pfam_name': lambda x: tuple(x.unique())})
    .rename(columns={'ID': 'all_counts'})
    .reset_index()
)

all_go_counts['p'] = all_go_counts['all_counts'] / all_go_prot_num

all_go_counts.head()
```

Out[41]:

|  | GO\_num | all\_counts | GO\_term | pfam\_name | p |
| --- | --- | --- | --- | --- | --- |
| 0 | GO:0000001 | 747 | mitochondrion inheritance | (MDM31\_MDM32,) | 0.000273 |
| 1 | GO:0000002 | 752 | mitochondrial genome maintenance | (Mgm101p, GEP5, RRG8) | 0.000275 |
| 2 | GO:0000030 | 5428 | mannosyltransferase activity | (Glyco\_transf\_15, PMT, DUF3589) | 0.001987 |
| 3 | GO:0000045 | 583 | autophagosome assembly | (APG12,) | 0.000213 |
| 4 | GO:0000049 | 2597 | tRNA binding | (Ribonuclease\_P, tRNA-synt\_2d, tRNA\_bind, CTU2) | 0.000951 |

In [42]:

```
neigh_go = pd.merge(go, neigh_pfam)
```

In [43]:

```
indices_to_keep = []

for idx, row in neigh_go.iterrows():
    if row['pfam_ID'] not in domains_filter['T1'][row['T1']]:
        indices_to_keep.append(idx)
```

In [44]:

```
# group records by TE type (with/without domain, TE family)
counts = (
    neigh_go
    .loc[indices_to_keep]                             # discard records with TE-related proteins
    .query('T2 in @T2s')
    .groupby(['domain', 'T2', 'GO_num'])
    .agg({'ID': lambda x: len(x),                     # count proteins with given GO and TE of given type
          'GO_term': lambda x: x.unique()[0],         # get GO term
          'pfam_name': lambda x: tuple(x.unique()),   # get pfam names
          'class': lambda x: tuple(x.unique())})      # get taxonomic info
    .rename(columns={'ID': 'counts'})
    .dropna(subset=['counts'])
    .reset_index()
)

assert not counts.duplicated().any()

# get total number of proteins with TE insterted
prot_nums = counts.groupby(['domain', 'T2'])['counts'].sum()

count_table = pd.merge(
    counts,
    all_go_counts,
    on=['GO_num'],
    how='right',
    suffixes=('s', 's_all')
).dropna(subset=['T2']).fillna({'counts': 0})

index = list(zip(count_table['domain'], count_table['T2']))
count_table['prot_num'] = prot_nums.loc[index].values.astype(int)

fill_count_table(count_table)

assert (count_table['prot_num'] > 0).all()
assert not count_table.duplicated().any()
assert not count_table.isnull().any().any()
```

In [45]:

```
hits = count_table.query('pval_binom_test < 0.05')
hits['sort_key'] = hits['fold_change'].abs()
del hits['GO_terms_all']
```

In [46]:

```
for (domain, T2), hits_tab in hits.groupby(['domain', 'T2']):
    domain = 'domain' if domain else 'no_domain'
    print(domain, T2, len(hits_tab))

    if len(hits_tab) == 0:
        continue

    hits_tab['pfam_names'] = hits_tab['pfam_names'].str.join(', ')
    hits_tab['pfam_names_all'] = hits_tab['pfam_names_all'].str.join(', ')
    hits_tab['class'] = hits_tab['class'].str.join(', ')
    hits_tab = hits_tab.sort_values(['all_counts', 'sort_key'], ascending=False)

    wks_name = '%s_%s_GO' % (T2, domain)
    hits_tab.to_excel(excel_writer, sheet_name=wks_name)

excel_writer.save()
```

```
no_domain ACADEM 7
no_domain CMC 55
no_domain COPIA 86
no_domain CRYPTON 46
no_domain DIRS 40
no_domain GYPSY 95
no_domain HAT 62
no_domain HELITRON 65
no_domain L1 49
no_domain MULE 50
no_domain PIF 41
no_domain TAD1 66
no_domain TCMAR 133
no_domain ZISUPTON 29
domain ACADEM 8
domain COPIA 6
domain GYPSY 33
domain HAT 4
domain L1 3
domain TCMAR 10
```

# TE insertion into genes vs lifestyle¶

In [47]:

```
agg_fun = {feature: np.unique for feature in lifestyle + ['genes_in_genome_fraction', 'subphylum']}
agg_fun['ID'] = lambda x: (~x.isnull()).mean()
```

In [48]:

```
lifestyle_neigh = (
    pd.merge(neighbours, te_fraction.dropna(subset=lifestyle))
    .groupby(['assembly_ID', 'domain'])
    .agg(agg_fun)
    .reset_index()
    .rename(columns={'ID': 'TEs_in_gene'})
)
```

In [49]:

```
lifestyle_neigh.head()
```

Out[49]:

|  | assembly\_ID | domain | animal | fungus | plant | pathogen | saprotroph | symbiont | soil/dung | water | genes\_in\_genome\_fraction | subphylum | TEs\_in\_gene |
| --- | --- | --- | --- | --- | --- | --- | --- | --- | --- | --- | --- | --- | --- |
| 0 | GCA\_000001985.1 | False | 1.0 | 0.0 | 0.0 | 1.0 | 1.0 | 0.0 | 1.0 | 0.0 | 0.661152 | Pezizomycotina | 0.769045 |
| 1 | GCA\_000001985.1 | True | 1.0 | 0.0 | 0.0 | 1.0 | 1.0 | 0.0 | 1.0 | 0.0 | 0.661152 | Pezizomycotina | 0.255521 |
| 2 | GCA\_000002495.2 | False | 0.0 | 0.0 | 1.0 | 1.0 | 0.0 | 0.0 | 0.0 | 0.0 | 0.615452 | Pezizomycotina | 0.695004 |
| 3 | GCA\_000002495.2 | True | 0.0 | 0.0 | 1.0 | 1.0 | 0.0 | 0.0 | 0.0 | 0.0 | 0.615452 | Pezizomycotina | 0.078613 |
| 4 | GCA\_000002515.1 | False | 0.0 | 0.0 | 1.0 | 0.0 | 1.0 | 0.0 | 1.0 | 0.0 | 0.707457 | Saccharomycotina | 0.898420 |

In [50]:

```
Ns = lifestyle_neigh.query('domain')[lifestyle].apply(pd.value_counts)
Ns
```

Out[50]:

|  | animal | fungus | plant | pathogen | saprotroph | symbiont | soil/dung | water |
| --- | --- | --- | --- | --- | --- | --- | --- | --- |
| 0.0 | 355 | 598 | 290 | 255 | 236 | 576 | 410 | 600 |
| 1.0 | 254 | 11 | 319 | 354 | 373 | 33 | 199 | 9 |

In [51]:

```
to_plot = [
    'animal',
    'soil/dung',
    'saprotroph',
    'symbiont',
    'plant',
    'pathogen',
]
```

In [52]:

```
fig, axs = plt.subplots(ncols=2, nrows=3, figsize=(6, 9))
axs = axs.flatten()

for f, ax in zip(to_plot, axs):
    sns.boxplot(data=lifestyle_neigh, x='domain', y='TEs_in_gene', hue=f, ax=ax)
    ax.set_ylabel('fraction of TEs in genes')
    ax.set_xlabel('')
    ax.set_xticklabels(['no domain', 'domain'])
    ax.set_ylim(-0.05, 1.65)
    handles, labels = ax.get_legend_handles_labels()
    labels = ['not %s (n=%s)' % (f, Ns.loc[float(l), f]) if l == '0.0'
              else '%s (n=%s)' % (f, Ns.loc[float(l), f]) for l in labels]
    ax.legend(handles, labels, loc='upper left')
fig.tight_layout()

fig.savefig('Fig.4_lifestyle_in_genes.pdf')
```

## Distance between TEs and genes¶

In [53]:

```
not_inserted = pd.merge(neighbours, te_fraction.dropna(subset=lifestyle))
not_inserted = not_inserted[not_inserted['ID'].isnull()]
```

In [54]:

```
len(not_inserted)
```

Out[54]:

```
1223746
```

In [55]:

```
not_inserted.head()
```

Out[55]:

|  | assembly\_ID | contig\_ID | TE\_start | TE\_end | L\_type | L\_ID | L\_localization | L\_start | L\_end | R\_type | ... | saprotroph | symbiont | animal | fungus | plant | soil/dung | water | TEs\_in\_gene | TEs\_in\_gene\_fraction | genes\_in\_genome\_fraction |
| --- | --- | --- | --- | --- | --- | --- | --- | --- | --- | --- | --- | --- | --- | --- | --- | --- | --- | --- | --- | --- | --- |
| 0 | GCA\_000001985.1 | DS995903.1 | 1834 | 2309 | gene | EEA21206.1 | Other | 28 | 838 | te | ... | 1.0 | 0.0 | 1.0 | 0.0 | 0.0 | 1.0 | 0.0 | 1908 | 0.769045 | 0.661152 |
| 1 | GCA\_000001985.1 | DS995903.1 | 2585 | 2875 | te | DS995903.1 | NaN | 1834 | 2309 | NaN | ... | 1.0 | 0.0 | 1.0 | 0.0 | 0.0 | 1.0 | 0.0 | 1908 | 0.769045 | 0.661152 |
| 2 | GCA\_000001985.1 | DS995903.1 | 4146 | 9205 | NaN | NaN | NaN | -1000 | -1000 | NaN | ... | 1.0 | 0.0 | 1.0 | 0.0 | 0.0 | 1.0 | 0.0 | 1908 | 0.769045 | 0.661152 |
| 3 | GCA\_000001985.1 | DS995903.1 | 11377 | 11630 | NaN | NaN | NaN | -1000 | -1000 | gene | ... | 1.0 | 0.0 | 1.0 | 0.0 | 0.0 | 1.0 | 0.0 | 1908 | 0.769045 | 0.661152 |
| 6 | GCA\_000001985.1 | DS995903.1 | 14300 | 14538 | gene | EEA21209.1 | Other | 11766 | 14126 | gene | ... | 1.0 | 0.0 | 1.0 | 0.0 | 0.0 | 1.0 | 0.0 | 1908 | 0.769045 | 0.661152 |

5 rows × 53 columns

In [56]:

```
L_dist = not_inserted[not_inserted['L_type'] == 'gene']
L_dist['TE_gene_dist'] = L_dist['TE_start'] - L_dist['L_end']

R_dist = not_inserted[not_inserted['R_type'] == 'gene']
R_dist['TE_gene_dist'] = R_dist['R_start'] - R_dist['TE_end']
```

In [57]:

```
for lifestle_group in to_plot:
    TE_gene_dist = pd.concat((L_dist[[lifestle_group, 'TE_gene_dist']],
                              R_dist[[lifestle_group, 'TE_gene_dist']])).set_index(lifestle_group)

    print(TE_gene_dist.groupby(lifestle_group).agg('mean').sort_index())
    print('pvalue', scipy.stats.mannwhitneyu(TE_gene_dist.loc[0], TE_gene_dist.loc[1]).pvalue)
    print()
```

```
        TE_gene_dist
animal              
0.0       364.994437
1.0       357.103944
pvalue 5.43284420693e-11

           TE_gene_dist
soil/dung              
0.0          364.465639
1.0          360.186938
pvalue 1.7184015265e-06

            TE_gene_dist
saprotroph              
0.0           391.119941
1.0           342.573550
pvalue 0.0

          TE_gene_dist
symbiont              
0.0         367.935728
1.0         331.719060
pvalue 3.87165582186e-260

       TE_gene_dist
plant              
0.0      351.172558
1.0      369.800039
pvalue 7.69366292173e-78

          TE_gene_dist
pathogen              
0.0         339.342529
1.0         384.156309
pvalue 0.0
```

# SSP¶

In [58]:

```
plant_assemblies = set(fungi_data.query('plant == 1')['assembly_ID'])
plant_pathogen_assemblies = set(fungi_data.query('plant == 1 and pathogen == 1')['assembly_ID'])

len(plant_assemblies), len(plant_pathogen_assemblies)
```

Out[58]:

```
(319, 140)
```

In [59]:

```
prot_info['ssp'] = (
    (prot_info['aas_num'] < 300)
    & (prot_info['secreted'])
    # & (prot_info['cys_num'] / prot_info['aas_num'] > 0.05)
    # uncomment the line above to restrict SSPs to proteins having more than 5% of cysteins
)
```

In [60]:

```
print('%s SSPs in analysed genomes (%d%% of all proteins)'
      % (prot_info['ssp'].sum(), 100*prot_info['ssp'].mean()))

print(prot_info.query('assembly_ID in @plant_assemblies')['ssp'].sum(),
      'SSPs in genomes of organisms with a plant host')

print(prot_info.query('assembly_ID in @plant_pathogen_assemblies')['ssp'].sum(),
      'SSPs in plant pathogens\' genomes')
```

```
397079 SSPs in analysed genomes (6% of all proteins)
251870 SSPs in genomes of organisms with a plant host
116789 SSPs in plant pathogens' genomes
```

In [61]:

```
# gene on either side or TE inserted into gene
neigh_any = pd.concat((
    neighbours[['assembly_ID', 'domain', 'T1', 'ID']].dropna(),
    neighbours[['assembly_ID', 'domain', 'T1', 'L_ID']].dropna().rename(columns={'L_ID': 'ID'}),
    neighbours[['assembly_ID', 'domain', 'T1', 'R_ID']].dropna().rename(columns={'R_ID': 'ID'})
)).drop_duplicates()
```

In [62]:

```
neigh_prot_info = prot_info.loc[prot_info['ID'].isin(set(neigh_any['ID'].unique()))]
print('%s SSPs in TE neighbourhood (%d%% of all proteins in TE neighbourhood)'
      % (neigh_prot_info['ssp'].sum(), 100*neigh_prot_info['ssp'].mean()))
```

```
38101 SSPs in TE neighbourhood (3% of all proteins in TE neighbourhood)
```

In [63]:

```
ssp_per_assembly = neigh_prot_info.groupby('assembly_ID')['ssp'].agg('sum')
ssp_per_assembly = pd.merge(ssp_per_assembly.reset_index(), fungi_data)
ssp_per_assembly = pd.merge(ssp_per_assembly, prot_info.groupby('assembly_ID')['ssp'].agg('sum').reset_index(),
                            on='assembly_ID', suffixes=('_neigh', '_total'))
```

In [64]:

```
ssp_neigh_per_class = (
    ssp_per_assembly
    .groupby('class')
    .agg({'ssp_neigh': ['size', 'mean', 'sum'], 'ssp_total': ['mean', 'sum']})
)

ssp_neigh_per_class['ssp_neigh_fraction'] = (
    ssp_neigh_per_class[('ssp_neigh', 'sum')]
    / ssp_neigh_per_class[('ssp_total', 'sum')]
)

del ssp_neigh_per_class[('ssp_neigh', 'sum')]
del ssp_neigh_per_class[('ssp_total', 'sum')]
ssp_neigh_per_class.columns = ['num_assemblies', 'mean_ssp_neigh', 'mean_ssp_total', 'ssp_neigh_fraction']

ssp_neigh_per_class.to_excel(excel_writer, sheet_name='TE_SSP')
excel_writer.save()
ssp_neigh_per_class
```

Out[64]:

|  | num\_assemblies | mean\_ssp\_neigh | mean\_ssp\_total | ssp\_neigh\_fraction |
| --- | --- | --- | --- | --- |
| class |  |  |  |  |
| Agaricomycetes | 72 | 92.472222 | 1155.194444 | 0.080049 |
| Blastocladiomycetes | 1 | 17.000000 | 991.000000 | 0.017154 |
| Chytridiomycetes | 3 | 107.333333 | 608.000000 | 0.176535 |
| Cryptomycota | 1 | 9.000000 | 400.000000 | 0.022500 |
| Dacrymycetes | 3 | 41.333333 | 861.666667 | 0.047969 |
| Dothideomycetes | 39 | 69.333333 | 820.794872 | 0.084471 |
| Entomophthoromycetes | 1 | 323.000000 | 1466.000000 | 0.220327 |
| Eurotiomycetes | 122 | 39.147541 | 525.213115 | 0.074536 |
| Exobasidiomycetes | 6 | 25.333333 | 521.833333 | 0.048547 |
| Glomeromycetes | 2 | 392.500000 | 1712.500000 | 0.229197 |
| Leotiomycetes | 33 | 102.848485 | 632.575758 | 0.162587 |
| Malasseziomycetes | 3 | 6.666667 | 145.333333 | 0.045872 |
| Microbotryomycetes | 5 | 8.200000 | 243.400000 | 0.033689 |
| Microsporidia | 26 | 36.384615 | 309.115385 | 0.117706 |
| Mixiomycetes | 2 | 1.000000 | 497.000000 | 0.002012 |
| Monoblepharidomycetes | 1 | 16.000000 | 843.000000 | 0.018980 |
| Orbiliomycetes | 3 | 17.333333 | 617.666667 | 0.028063 |
| Pezizomycetes | 1 | 58.000000 | 487.000000 | 0.119097 |
| Pneumocystidomycetes | 4 | 9.500000 | 173.250000 | 0.054834 |
| Pucciniomycetes | 5 | 562.200000 | 1579.000000 | 0.356048 |
| Saccharomycetes | 106 | 16.584906 | 294.990566 | 0.056222 |
| Schizosaccharomycetes | 4 | 9.000000 | 223.500000 | 0.040268 |
| Sordariomycetes | 122 | 78.090164 | 804.204918 | 0.097102 |
| Taphrinomycetes | 1 | 19.000000 | 216.000000 | 0.087963 |
| Tremellomycetes | 31 | 10.000000 | 348.193548 | 0.028720 |
| Ustilaginomycetes | 9 | 15.222222 | 304.333333 | 0.050018 |
| Wallemiomycetes | 2 | 6.000000 | 262.000000 | 0.022901 |
| Xylonomycetes | 1 | 19.000000 | 502.000000 | 0.037849 |

## SSPs with TE in neighbourhood¶

In [65]:

```
for prot_tab in (prot_info, prot_info.query('assembly_ID in @plant_assemblies'),
                 prot_info.query('assembly_ID in @plant_pathogen_assemblies')):

    all_counts = (
        prot_tab
        .groupby('ssp')['ID']
        .size()
        .reset_index()
        .rename(columns={'ID': 'all_counts'})
    )

    all_counts['p'] = all_counts['all_counts'] / len(prot_tab)
    
    neigh_ssp = pd.merge(neigh_any, prot_tab)

    counts = (
        neigh_ssp
        .groupby(['domain', 'ssp'])
        .agg({'ID': lambda x: len(x.unique())})
        .reset_index()
        .rename(columns={'ID': 'counts'})
        .dropna(subset=['counts'])
    )

    # number of proteins in TEs neighbourhood
    prot_nums = counts.groupby('domain')['counts'].sum()

    count_table = pd.merge(counts, all_counts, on=['ssp'], how='right').fillna({'counts': 0})
    count_table['prot_num'] = prot_nums.iloc[[0,1,0,1]].values

    fill_count_table(count_table)
    print(count_table)
```

```
   domain    ssp  counts  all_counts         p  prot_num  observed_freq  \
0   False  False  952155     6064003  0.938543    987299       0.964404   
1    True  False   83127     6064003  0.938543     86699       0.958800   
2   False   True   35144      397079  0.061457    987299       0.035596   
3    True   True    3572      397079  0.061457     86699       0.041200   

   expected_counts        score  expected_score  fold_change  pval_binom_test  
0           926623 -6694.974827       -6.393881     1.027554     0.000000e+00  
1            81371  -351.628544       -5.177615     1.021583     1.254507e-31  
2            60676 -6694.974827       -6.393881    -1.726510     0.000000e+00  
3             5328  -351.628544       -5.177615    -1.491675     1.254507e-31  
   domain    ssp  counts  all_counts         p  prot_num  observed_freq  \
0   False  False  560438     3646933  0.935398    582584       0.961987   
1    True  False   53734     3646933  0.935398     56276       0.954830   
2   False   True   22146      251870  0.064602    582584       0.038013   
3    True   True    2542      251870  0.064602     56276       0.045170   

   expected_counts        score  expected_score  fold_change  pval_binom_test  
0           544948 -3969.888742       -6.153407     1.028425     0.000000e+00  
1            52640  -200.091258       -4.984917     1.020774     3.492898e-19  
2            37636 -3969.888742       -6.153407    -1.699450     0.000000e+00  
3             3636  -200.091258       -4.984917    -1.430187     3.492898e-19  
   domain    ssp  counts  all_counts         p  prot_num  observed_freq  \
0   False  False  275730     1677292  0.934903    287595       0.958744   
1    True  False   24883     1677292  0.934903     26204       0.949588   
2   False   True   11865      116789  0.065097    287595       0.041256   
3    True   True    1321      116789  0.065097     26204       0.050412   

   expected_counts        score  expected_score  fold_change  pval_binom_test  
0           268873 -1537.402133       -5.804020     1.025501    1.036195e-142  
1            24498   -54.584710       -4.606294     1.015707     1.235731e-05  
2            18722 -1537.402133       -5.804020    -1.577878    1.036195e-142  
3             1706   -54.584710       -4.606294    -1.291293     1.235731e-05
```

### Divided by TE type¶

In [66]:

```
for prot_tab in (prot_info, prot_info.query('assembly_ID in @plant_assemblies'),
                 prot_info.query('assembly_ID in @plant_pathogen_assemblies')):

    all_counts = (
        prot_tab
        .groupby('ssp')['ID']
        .size()
        .reset_index()
        .rename(columns={'ID': 'all_counts'})
    )

    all_counts['p'] = all_counts['all_counts'] / len(prot_tab)
    
    neigh_ssp = pd.merge(neigh_any, prot_tab)

    counts = (
        neigh_ssp
        .query('T1 in @T1s')
        .groupby(['domain', 'T1', 'ssp'])
        .agg({'ID': lambda x: len(x.unique())})
        .rename(columns={'ID': 'counts'})
        .dropna(subset=['counts'])
        .reset_index()
    )

    # number of proteins in TEs neighbourhood
    prot_nums = counts.groupby(['domain', 'T1'])['counts'].sum()

    count_table = pd.merge(counts, all_counts, on=['ssp'], how='right').dropna(subset=['T1']).fillna({'counts': 0})
    count_table['prot_num'] = prot_nums.loc[list(zip(count_table['domain'], count_table['T1']))].values

    fill_count_table(count_table)
    print(count_table)
```

```
    domain    T1    ssp  counts  all_counts         p  prot_num  \
0    False   DNA  False  510158     6064003  0.938543    527408   
1    False  LINE  False  132335     6064003  0.938543    136952   
2    False   LTR  False  303397     6064003  0.938543    315654   
3    False    RC  False   27017     6064003  0.938543     27816   
4     True   DNA  False   44415     6064003  0.938543     46073   
5     True  LINE  False    8955     6064003  0.938543      9357   
6     True   LTR  False   20619     6064003  0.938543     21671   
7     True    RC  False    2790     6064003  0.938543      2940   
8    False   DNA   True   17250      397079  0.061457    527408   
9    False  LINE   True    4617      397079  0.061457    136952   
10   False   LTR   True   12257      397079  0.061457    315654   
11   False    RC   True     799      397079  0.061457     27816   
12    True   DNA   True    1658      397079  0.061457     46073   
13    True  LINE   True     402      397079  0.061457      9357   
14    True   LTR   True    1052      397079  0.061457     21671   
15    True    RC   True     150      397079  0.061457      2940   

    observed_freq  expected_counts        score  expected_score  fold_change  \
0        0.967293           494995 -4518.260856       -6.080385     1.030633   
1        0.966287           128535 -1088.038471       -5.406245     1.029561   
2        0.961170           296255 -1605.587472       -5.823719     1.024108   
3        0.971276            26107  -322.720407       -4.609190     1.034876   
4        0.964014            43241  -306.540634       -4.861663     1.027139   
5        0.957038             8782   -34.722487       -4.064561     1.019706   
6        0.951456            20339   -37.995238       -4.484513     1.013758   
7        0.948980             2759    -6.335400       -3.487173     1.011120   
8        0.032707            32413 -4518.260856       -6.080385    -1.879011   
9        0.033713             8417 -1088.038471       -5.406245    -1.822973   
10       0.038830            19399 -1605.587472       -5.823719    -1.582701   
11       0.028724             1709  -322.720407       -4.609190    -2.139536   
12       0.035986             2832  -306.540634       -4.861663    -1.707787   
13       0.042962              575   -34.722487       -4.064561    -1.430481   
14       0.048544             1332   -37.995238       -4.484513    -1.266003   
15       0.051020              181    -6.335400       -3.487173    -1.204558   

    pval_binom_test  
0      0.000000e+00  
1      2.713383e-92  
2     4.303836e-141  
3      1.299418e-26  
4      1.090908e-26  
5      1.659735e-03  
6      7.267109e-04  
7      5.270450e-01  
8      0.000000e+00  
9      2.713383e-92  
10    4.303836e-141  
11     1.299418e-26  
12     1.090908e-26  
13     1.659735e-03  
14     7.267109e-04  
15     5.270450e-01  
    domain    T1    ssp  counts  all_counts         p  prot_num  \
0    False   DNA  False  295163     3646933  0.935398    305961   
1    False  LINE  False   75279     3646933  0.935398     78077   
2    False   LTR  False  188461     3646933  0.935398    196710   
3    False    RC  False   14788     3646933  0.935398     15145   
4     True   DNA  False   28063     3646933  0.935398     29221   
5     True  LINE  False    5668     3646933  0.935398      5962   
6     True   LTR  False   14647     3646933  0.935398     15450   
7     True    RC  False    1846     3646933  0.935398      1945   
8    False   DNA   True   10798      251870  0.064602    305961   
9    False  LINE   True    2798      251870  0.064602     78077   
10   False   LTR   True    8249      251870  0.064602    196710   
11   False    RC   True     357      251870  0.064602     15145   
12    True   DNA   True    1158      251870  0.064602     29221   
13    True  LINE   True     294      251870  0.064602      5962   
14    True   LTR   True     803      251870  0.064602     15450   
15    True    RC   True      99      251870  0.064602      1945   

    observed_freq  expected_counts        score  expected_score  fold_change  \
0        0.964708           286195 -2583.943540       -5.831413     1.031334   
1        0.964164            73033  -636.172459       -5.148541     1.030752   
2        0.958065           184002  -953.221338       -5.610548     1.024233   
3        0.976428            14167  -278.755225       -4.328487     1.043863   
4        0.960371            27333  -177.919861       -4.657244     1.026698   
5        0.950688             5577   -16.232604       -3.862434     1.016346   
6        0.948026            14452   -25.995863       -4.338516     1.013500   
7        0.949100             1819    -6.435949       -3.304775     1.014648   
8        0.035292            19766 -2583.943540       -5.831413    -1.830492   
9        0.035836             5044  -636.172459       -5.148541    -1.802688   
10       0.041935            12708  -953.221338       -5.610548    -1.540530   
11       0.023572              978  -278.755225       -4.328487    -2.740604   
12       0.039629             1888  -177.919861       -4.657244    -1.630165   
13       0.049312              385   -16.232604       -3.862434    -1.310056   
14       0.051974              998   -25.995863       -4.338516    -1.242963   
15       0.050900              126    -6.435949       -3.304775    -1.269199   

    pval_binom_test  
0     5.010909e-229  
1      7.671012e-57  
2      1.632605e-88  
3      7.156815e-23  
4      1.483520e-16  
5      6.157594e-02  
6      7.245816e-03  
7      4.817443e-01  
8     5.010909e-229  
9      7.671012e-57  
10     1.632605e-88  
11     7.156815e-23  
12     1.483520e-16  
13     6.157594e-02  
14     7.245816e-03  
15     4.817443e-01  
    domain    T1    ssp  counts  all_counts         p  prot_num  \
0    False   DNA  False  150486     1677292  0.934903    156666   
1    False  LINE  False   39060     1677292  0.934903     40608   
2    False   LTR  False   87390     1677292  0.934903     91479   
3    False    RC  False    5805     1677292  0.934903      5948   
4     True   DNA  False   12922     1677292  0.934903     13594   
5     True  LINE  False    2878     1677292  0.934903      3041   
6     True   LTR  False    6418     1677292  0.934903      6764   
7     True    RC  False     750     1677292  0.934903       778   
8    False   DNA   True    6180      116789  0.065097    156666   
9    False  LINE   True    1548      116789  0.065097     40608   
10   False   LTR   True    4089      116789  0.065097     91479   
11   False    RC   True     143      116789  0.065097      5948   
12    True   DNA   True     672      116789  0.065097     13594   
13    True  LINE   True     163      116789  0.065097      3041   
14    True   LTR   True     346      116789  0.065097      6764   
15    True    RC   True      28      116789  0.065097       778   

    observed_freq  expected_counts       score  expected_score  fold_change  \
0        0.960553           146468 -982.675392       -5.500277     1.027436   
1        0.961879            37965 -287.310011       -4.825196     1.028855   
2        0.955301            85524 -354.081251       -5.231290     1.021818   
3        0.975958             5561 -110.429396       -3.864782     1.043914   
4        0.950566            12709  -33.883637       -4.278171     1.016754   
5        0.946399             2843   -6.939443       -3.529836     1.012297   
6        0.948847             6324  -15.427156       -3.929005     1.014915   
7        0.964010              727   -8.969954       -2.853918     1.031134   
8        0.039447            10198 -982.675392       -5.500277    -1.650236   
9        0.038121             2643 -287.310011       -4.825196    -1.707657   
10       0.044699             5955 -354.081251       -5.231290    -1.456345   
11       0.024042              387 -110.429396       -3.864782    -2.707664   
12       0.049434              885  -33.883637       -4.278171    -1.316855   
13       0.053601              198   -6.939443       -3.529836    -1.214475   
14       0.051153              440  -15.427156       -3.929005    -1.272587   
15       0.035990               51   -8.969954       -2.853918    -1.808762   

    pval_binom_test  
0      2.964510e-90  
1      2.148505e-26  
2      8.693828e-34  
3      1.958665e-09  
4      1.268088e-03  
5      4.476284e-01  
6      7.206437e-02  
7      2.675948e-01  
8      2.964510e-90  
9      2.148505e-26  
10     8.693828e-34  
11     1.958665e-09  
12     1.268088e-03  
13     4.476284e-01  
14     7.206437e-02  
15     2.675948e-01
```

## SSPs with TE insterted, diveded by TE type¶

In [67]:

```
for prot_tab in (prot_info, prot_info.query('assembly_ID in @plant_assemblies'),
                 prot_info.query('assembly_ID in @plant_pathogen_assemblies')):

    all_counts = (
        prot_tab
        .groupby('ssp')['ID']
        .size()
        .reset_index()
        .rename(columns={'ID': 'all_counts'})
    )
    all_counts['p'] = all_counts['all_counts'] / len(prot_tab)

    neigh_ssp = pd.merge(neighbours, prot_tab)
    counts = (
        neigh_ssp
        .query('T1 in @T1s')
        .groupby(['domain', 'T1', 'ssp'])
        .agg({'ID': lambda x: len(x.unique())})
        .rename(columns={'ID': 'counts'})
        .dropna(subset=['counts'])
        .reset_index()
    )

    # number of proteins in TEs neighbourhood
    prot_nums = counts.groupby(['domain', 'T1'])['counts'].sum()

    count_table = pd.merge(counts, all_counts, on=['ssp'], how='right').dropna(subset=['T1']).fillna({'counts': 0})
    count_table['prot_num'] = prot_nums.loc[list(zip(count_table['domain'], count_table['T1']))].values

    fill_count_table(count_table)
    print(count_table)
```

```
    domain    T1    ssp  counts  all_counts         p  prot_num  \
0    False   DNA  False  402028     6064003  0.938543    409816   
1    False  LINE  False  103936     6064003  0.938543    106249   
2    False   LTR  False  212023     6064003  0.938543    216451   
3    False    RC  False   19058     6064003  0.938543     19268   
4     True   DNA  False   22778     6064003  0.938543     22882   
5     True  LINE  False    3974     6064003  0.938543      4044   
6     True   LTR  False    6044     6064003  0.938543      6113   
7     True    RC  False     927     6064003  0.938543       934   
8    False   DNA   True    7788      397079  0.061457    409816   
9    False  LINE   True    2313      397079  0.061457    106249   
10   False   LTR   True    4428      397079  0.061457    216451   
11   False    RC   True     210      397079  0.061457     19268   
12    True   DNA   True     104      397079  0.061457     22882   
13    True  LINE   True      70      397079  0.061457      4044   
14    True   LTR   True      69      397079  0.061457      6113   
15    True    RC   True       7      397079  0.061457       934   

    observed_freq  expected_counts        score  expected_score  fold_change  \
0        0.980996           384630 -8650.320451       -5.954251     1.045233   
1        0.978230            99719 -1908.969953       -5.279323     1.042286   
2        0.979543           203149 -4199.823045       -5.635075     1.043684   
3        0.989101            18084  -640.289038       -4.425638     1.053869   
4        0.995455            21476 -1073.361655       -4.511568     1.060639   
5        0.982690             3795   -97.007101       -3.646695     1.047038   
6        0.988713             5737  -200.842281       -3.852354     1.053455   
7        0.992505              877   -38.993788       -2.911942     1.057496   
8        0.019004            25186 -8650.320451       -5.954251    -3.233960   
9        0.021770             6530 -1908.969953       -5.279323    -2.823065   
10       0.020457            13302 -4199.823045       -5.635075    -3.004164   
11       0.010899             1184  -640.289038       -4.425638    -5.638830   
12       0.004545             1406 -1073.361655       -4.511568   -13.521732   
13       0.017310              249   -97.007101       -3.646695    -3.550461   
14       0.011287              376  -200.842281       -3.852354    -5.444738   
15       0.007495               57   -38.993788       -2.911942    -8.200126   

    pval_binom_test  
0      0.000000e+00  
1     4.364699e-146  
2     9.205537e-317  
3      1.662446e-43  
4      4.219070e-65  
5      1.425618e-07  
6      4.320874e-14  
7      4.341634e-03  
8      0.000000e+00  
9     4.364699e-146  
10    9.205537e-317  
11     1.662446e-43  
12     4.219070e-65  
13     1.425618e-07  
14     4.320874e-14  
15     4.341634e-03  
    domain    T1    ssp  counts  all_counts         p  prot_num  \
0    False   DNA  False  228719     3646933  0.935398    233485   
1    False  LINE  False   58003     3646933  0.935398     59325   
2    False   LTR  False  128251     3646933  0.935398    131148   
3    False    RC  False   11208     3646933  0.935398     11313   
4     True   DNA  False   14295     3646933  0.935398     14385   
5     True  LINE  False    2351     3646933  0.935398      2409   
6     True   LTR  False    3836     3646933  0.935398      3891   
7     True    RC  False     573     3646933  0.935398       578   
8    False   DNA   True    4766      251870  0.064602    233485   
9    False  LINE   True    1322      251870  0.064602     59325   
10   False   LTR   True    2897      251870  0.064602    131148   
11   False    RC   True     105      251870  0.064602     11313   
12    True   DNA   True      90      251870  0.064602     14385   
13    True  LINE   True      58      251870  0.064602      2409   
14    True   LTR   True      55      251870  0.064602      3891   
15    True    RC   True       5      251870  0.064602       578   

    observed_freq  expected_counts        score  expected_score  fold_change  \
0        0.979588           218401 -5071.768948       -5.696253     1.047241   
1        0.977716            55492 -1163.856916       -5.011300     1.045240   
2        0.977910           122676 -2596.236891       -5.407830     1.045448   
3        0.990719            10582  -443.510433       -4.182879     1.059141   
4        0.993743            13456  -657.996573       -4.302754     1.062375   
5        0.975924             2253   -45.402524       -3.411409     1.043324   
6        0.985865             3640  -120.910608       -3.648942     1.053952   
7        0.991349              541   -24.971211       -2.695241     1.059816   
8        0.020412            15084 -5071.768948       -5.696253    -3.164828   
9        0.022284             3833 -1163.856916       -5.011300    -2.899022   
10       0.022090             8472 -2596.236891       -5.407830    -2.924545   
11       0.009281              731  -443.510433       -4.182879    -6.960391   
12       0.006257              929  -657.996573       -4.302754   -10.325534   
13       0.024076              156   -45.402524       -3.411409    -2.683206   
14       0.014135              251  -120.910608       -3.648942    -4.570289   
15       0.008651               37   -24.971211       -2.695241    -7.467977   

    pval_binom_test  
0      0.000000e+00  
1      5.269516e-93  
2     1.330306e-206  
3      8.469154e-31  
4      2.923742e-43  
5      3.660726e-04  
6      2.467952e-09  
7      2.681158e-02  
8      0.000000e+00  
9      5.269516e-93  
10    1.330306e-206  
11     8.469154e-31  
12     2.923742e-43  
13     3.660726e-04  
14     2.467952e-09  
15     2.681158e-02  
    domain    T1    ssp  counts  all_counts         p  prot_num  \
0    False   DNA  False  115018     1677292  0.934903    117776   
1    False  LINE  False   28715     1677292  0.934903     29322   
2    False   LTR  False   59868     1677292  0.934903     61319   
3    False    RC  False    4542     1677292  0.934903      4590   
4     True   DNA  False    5391     1677292  0.934903      5429   
5     True  LINE  False    1206     1677292  0.934903      1250   
6     True   LTR  False    1874     1677292  0.934903      1900   
7     True    RC  False     279     1677292  0.934903       280   
8    False   DNA   True    2758      116789  0.065097    117776   
9    False  LINE   True     607      116789  0.065097     29322   
10   False   LTR   True    1451      116789  0.065097     61319   
11   False    RC   True      48      116789  0.065097      4590   
12    True   DNA   True      38      116789  0.065097      5429   
13    True  LINE   True      44      116789  0.065097      1250   
14    True   LTR   True      26      116789  0.065097      1900   
15    True    RC   True       1      116789  0.065097       280   

    observed_freq  expected_counts        score  expected_score  fold_change  \
0        0.976583           110109 -2201.767179       -5.357635     1.044582   
1        0.979299            27413  -640.881950       -4.662504     1.047487   
2        0.976337            57327 -1132.355658       -5.031339     1.044319   
3        0.989542             4291  -173.064820       -3.735836     1.058444   
4        0.993001             5076  -243.008756       -3.819049     1.062143   
5        0.964800             1169   -13.704811       -3.084605     1.031979   
6        0.986316             1776   -62.315845       -3.296441     1.054992   
7        0.996429              262   -15.877228       -2.337071     1.065809   
8        0.023417             7667 -2201.767179       -5.357635    -2.779857   
9        0.020701             1909  -640.881950       -4.662504    -3.144596   
10       0.023663             3992 -1132.355658       -5.031339    -2.750981   
11       0.010458              299  -173.064820       -3.735836    -6.224885   
12       0.006999              353  -243.008756       -3.819049    -9.300282   
13       0.035200               81   -13.704811       -3.084605    -1.849342   
14       0.013684              124   -62.315845       -3.296441    -4.757077   
15       0.003571               18   -15.877228       -2.337071   -18.227115   

    pval_binom_test  
0     1.941943e-178  
1      6.334525e-51  
2      3.665140e-92  
3      1.251747e-12  
4      1.212010e-16  
5      1.070400e-01  
6      4.342908e-05  
7      1.200544e-01  
8     1.941943e-178  
9      6.334525e-51  
10     3.665140e-92  
11     1.251747e-12  
12     1.212010e-16  
13     1.070400e-01  
14     4.342908e-05  
15     1.200544e-01
```

In [68]:

```
excel_writer.close()
```

In [ ]:

```

```
